# DMSO induces drastic changes in human cellular processes and epigenetic landscape *in vitro*

**DOI:** 10.1038/s41598-019-40660-0

**Published:** 2019-03-15

**Authors:** M. Verheijen, M. Lienhard, Y. Schrooders, O. Clayton, R. Nudischer, S. Boerno, B. Timmermann, N. Selevsek, R. Schlapbach, H. Gmuender, S. Gotta, J. Geraedts, R. Herwig, J. Kleinjans, F. Caiment

**Affiliations:** 1 Toxicogenomics, Maastricht University, Maastricht, Netherlands; 2 Computational Molecular Biology, Max-Planck-Institute for Molecular Genetics, Berlin, Germany; 3 F. Hoffmann-La Roche AG, Basel, Switzerland; 4 Functional Genomics Center Zurich, ETH Zurich and University of Zurich, Zurich, Switzerland; 5 Genedata AG, Basel, Switzerland; 6 Genetics and Cell Biology, Maastricht University, Medical Center, Maastricht, Netherlands

## Abstract

Though clinical trials for medical applications of dimethyl sulfoxide (DMSO) reported toxicity in the 1960s, later, the FDA classified DMSO in the safest solvent category. DMSO became widely used in many biomedical fields and biological effects were overlooked. Meanwhile, biomedical science has evolved towards sensitive high-throughput techniques and new research areas, including epigenomics and microRNAs. Considering its wide use, especially for cryopreservation and *in vitro* assays, we evaluated biological effect of DMSO using these technological innovations. We exposed 3D cardiac and hepatic microtissues to medium with or without 0.1% DMSO and analyzed the transcriptome, proteome and DNA methylation profiles. In both tissue types, transcriptome analysis detected >2000 differentially expressed genes affecting similar biological processes, thereby indicating consistent cross-organ actions of DMSO. Furthermore, microRNA analysis revealed large-scale deregulations of cardiac microRNAs and smaller, though still massive, effects in hepatic microtissues. Genome-wide methylation patterns also revealed tissue-specificity. While hepatic microtissues demonstrated non-significant changes, findings from cardiac microtissues suggested disruption of DNA methylation mechanisms leading to genome-wide changes. The extreme changes in microRNAs and alterations in the epigenetic landscape indicate that DMSO is not inert. Its use should be reconsidered, especially for cryopreservation of embryos and oocytes, since it may impact embryonic development.

## Introduction

Dimethyl sulfoxide (DMSO) is an organic polar aprotic molecule with an amphipathic nature that is ideal for dissolving poorly soluble polar and non-polar molecules. DMSO is widely used as solvent in toxicology and pharmacology, for cryopreservation of cells, and as penetration enhancer during topological treatments. The use of DMSO is so obvious that applied concentrations are often unreported. DMSO is generally accepted as nontoxic below 10% (v/v) and, in practice, it is assumed that effects of DMSO are negligible^1–3^.

Research from the 1960’s till 1990’s pinpointed a lot of biologically relevant effects of DMSO. It was even actively investigated for medical use, but because of adverse effects especially affecting the eyes, most clinical trials were halted in 1965 by the United States Food and Drug Administration (FDA). At this point in time, DMSO was seen as extremely toxic, comparable to thalidomide^4,5^. The effects of DMSO differ depending on dose and route of administration. Introducing DMSO concentrations higher than 50% into the blood resulted in instant hemolysis, white cell stacking and fibrinogen precipitation while direct injection of DMSO intravenously could cause local irritation and necrosis^6^. LD50 values gained from monkeys indicated that 880 grams applied on the skin or 320 grams injected intravenously would result in 50% mortality in 80 kg humans^7^. This relatively high dose changed the view on DMSO toxicity and the FDA classified DMSO in the same class as ethanol, namely class 3 solvent, which is the safest category with low toxic potential at levels normally accepted in pharmaceuticals^8^. This made the wide use of DMSO possible.

While generally used at relatively low concentrations, DMSO still has medically useful properties such as inducing anti-inflammation, nerve blockage (analgesia), diuretics, vasodilation and muscle relaxation^9^. Furthermore, in cell biology, DMSO is also used as inducer of cell differentiation, free radical scavenger and radioprotectant, but most often for cryopreservation. Cell cultures for research are often stored in liquid nitrogen using slow cooling methodology. To prevent damage by intracellular ice crystals, cells are slowly cooled to −80 degrees in the presence of 10% DMSO before storage in liquid nitrogen^3^. However, this procedure is insufficient for biomedical applications. Because slow cooling still induces damage due to extracellular ice crystals, cryopreservation of human oocytes and embryos for *in vitro* fertilization (IVF) is accomplished by vitrification, in which higher concentrations of cryoprotectants are used to prevent ice formation not only in the cells, but in the entire solution. Higher concentrations are achieved by using mixtures (for example 15% DMSO in combination with 15% ethylene glycol) which reduces the amount and toxicity of the individual cryoprotectants^10^. Furthermore, DMSO readily crosses most tissue membranes of lower animals and man^9^. For example, 2 hours after topological application, DMSO had penetrated all investigated hard and soft tissues (6 and 15 resp.) in rats^11^. DMSO is also able to enhance the permeability of other low molecular weight compounds, thereby making them pass membranes or going deeper into a tissue as they normally would^12^, a property highly useful in topological therapies. In 2009, the first to obtain FDA approval for topological DMSO usage was PENNSAID^®^, which contains diclofenac in a carrier with 45.5% DMSO^13,14^. This relatively high concentration of DMSO in topological applications is necessary because the skin is harder to penetrate than cell membranes.

Most insights into the molecular effects of DMSO were obtained last century often using high doses. In the meantime, biomedical science has evolved towards more sensitive high-throughput techniques and towards new areas of research, including epigenome modifications and microRNA-mediated gene silencing. Considering its wide use in many biological fields, we analyzed a relatively low dose of DMSO (0.1%, which is commonly applied in cell assays) to study the impact on the proteome, transcriptome and the epigenome. For this, we exposed *in vitro* 3D microtissues (a maturing iPSC-derived cardiac model and a mature hepatic model) to 0.1% DMSO and we collected samples in triplicate at 7 different time points during 2 weeks exposure (2 h, 8 h, 24 h, 72 h, 168 h, 240 h and 336 h). Thereafter, DMSO effects were assessed with full transcriptome analysis (using ribo-depleted total RNA sequencing and microRNA sequencing), whole-genome methylation profiling (using MeDIP-seq) and proteomics analysis (using mass spectrometry). Our analysis clearly demonstrated that DMSO cannot be considered biologically inert but induces large alterations in microRNAs (miRNA) and epigenetic landscape, especially in the maturing cardiac model.

## Results

**Figure 1:**
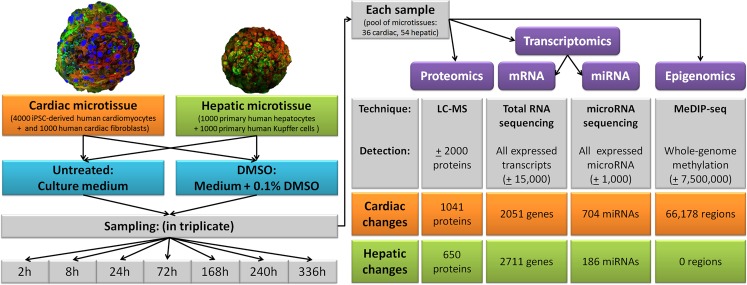
Graphical overview of experimental design combined with summary of differential entities of each analysis method. Tissue-specific information is depicted in orange for cardiac and green for hepatic. Furthermore, exposures are coloured blue and measurement platforms purple. Abbreviations: h = hours; mRNA = messenger RNA; miRNA = microRNA.

**Figure 2:**
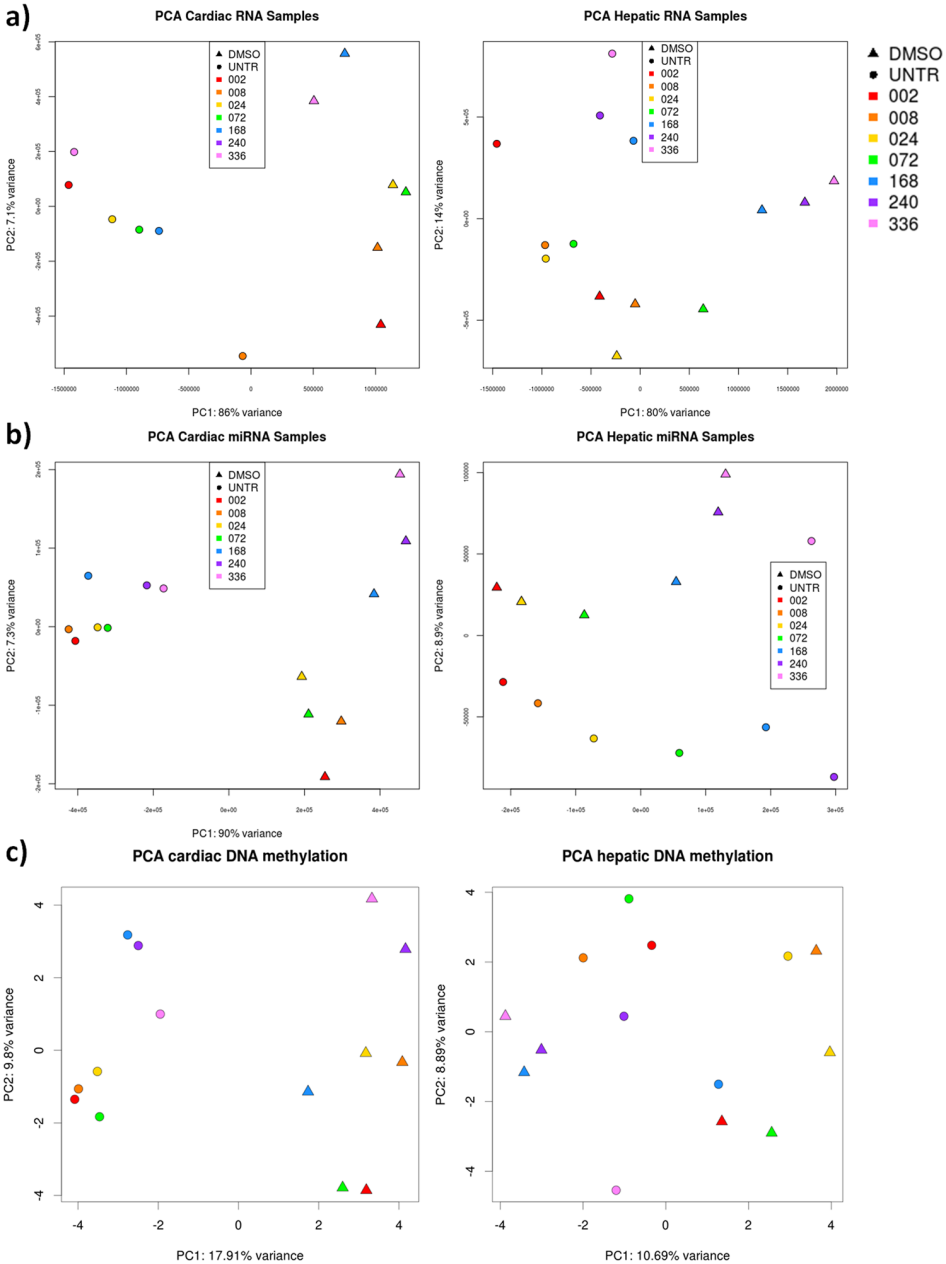
PCAs depicting differences between DMSO and UNTR for all measured platforms. (**a**) PCA of RNAs indicates clear differences in RNA expression between DMSO (triangle) and UNTR (circles). Cardiac samples (left) are more distinct from UNTR than hepatic samples (right). (**b**) PCA of miRNAs reveals clear separation between DMSO and UNTR in cardiac samples, while hepatic samples seem more susceptible to the duration of the exposure (as seen by colour pattern that corresponds to the specific time points, see legend). (**c**) PCA of promotor methylation indicates differences between DMSO and UNTR for cardiac samples but not for hepatic samples.

Human 3D microtissues (MTs) of a maturing cardiac model and a mature hepatic model were exposed to culture medium with or without 0.1% DMSO for two weeks with sampling time points at 2, 8, 72, 168, 240 and 336 hours. The proteome (approximately 2,000 measured proteins), the full transcriptome (including miRNAs) and whole-genome methylation were measured on material obtained from the same sample. Figure 1 contains a graphical overview of the experimental design. In order to obtain a first overview of DMSO-induced cross-omics effects, amounts of differentially changed entities (all corrected for multiple testing using FDR <0.05) are summarized for each platform. Numbers of differentially changed entities differed between the tissue types, with cardiac samples showing a larger effect of DMSO than hepatic, with the exception of mRNAs. This difference is especially noticeable for miRNAs and genome methylation. Because proteomics data was least informative due to its partial nature, these results were included in Supplementary Data. Furthermore, principal component analysis (PCA), using averages of triplicates, for each platform (Fig. 2 & Supplementary Data) depicts clear differences between 0.1% DMSO exposed (DMSO) and untreated (UNTR) samples, with the exception of methylation in hepatic MTs.

To study the molecular effects of DMSO, affected cellular processes were analysed using the full transcriptome. Thereafter, tissue-specific effects of DMSO on regulation of gene expression were investigated by analysing changes in miRNAs and genome-methylation.

### DMSO effects on cellular processes

DMSO effects on mRNAs were depicted by of PCA (Fig. 2a,b). The clear separation between UNTR and 0.1% DMSO indicated that DMSO was able to affect cellular processes by altering gene expression. Comparison between DMSO and UNTR resulted in 2051 differentially expressed genes (DEGs = FDR <0.05; of which 871 with |log2FC| >1) in cardiac MTs and 2711 DEGs (of which 1879 with |log2FC| >1) in hepatic MTs, of which 60.7% and 62.9% DEGs were downregulated respectively.

#### Pathway analysis of DEGs

To identify cellular processes affected by DMSO exposure, DEGs were used for pathway overrepresentation analysis using ConsensusPathDB^15^ with the curated Reactome database^16^. Significantly overrepresented pathways (q-value < 0.05) were ordered using the hierarchical connections between (sub-) pathways obtained from the Reactome Pathway Browser (Supplementary Tables 1,2). A summary containing the highest hierarchical pathway levels (from now on referred to as clusters) is included in Table 1 for cardiac and Table 2 for hepatic MTs.

**Table 1:** Pathway analysis of Cardiac DEGs & DEPs detected after 0.1% DMSO exposure.

Cluster name (stable identifier)	Set size	Ranking (DEGs)	Amount DEGs (%)	Amount DEGs (%)|log2FC| > 1	q-value	% DEGs Down-regulated
**Cellular responses to stress (R-HSA-2262752)**	**393**	**1**	**91 (23.5%)**	**28 (7.1%)**	**2.2E-09**	**61.5**
**Disease (R-HSA-1643685)**	**514**	**2**	**109 (21.5%)**	**33 (6.4%)**	**4.5E-09**	**61.5**
**Vesicle-mediated transport (R-HSA-5653656)**	**619**	**3**	**122 (20.0%)**	**48 (7.8%)**	**2.7E-08**	**55.7**
**Metabolism of proteins (R-HSA-392499)**	**1506**	**4**	**236 (16.0%)**	**80 (5.3%)**	**9.3E-07**	**68.2**
**Chromatin organization (R-HSA-4839726)**	**274**	**5**	**58 (21.3%)**	**19 (6.9%)**	**5.2E-05**	**53.4**
**Muscle contraction (R-HSA-397014)**	**198**	**6**	**41 (21.0%)**	**11 (5.6%)**	**9.5E-04**	**63.4**
**Gene Expression (R-HSA-74160)**	**1755**	**7**	**235 (13.7%)**	**76 (4.3%)**	**6.6E-03**	**55.7**
**Metabolism (R-HSA-1430728)**	**2035**	**8**	**269 (13.5%)**	**100 (4.9%)**	**7.9E-03**	**69.1**
**Extracellular matrix organization (R-HSA-1474244)**	**295**	**9**	**51 (17.6%)**	**23 (7.8%)**	**8.1E-03**	**52.9**
**Immune System (R-HSA-168256)**	**1950**	**10**	**257 (13.5%)**	**95 (4.9%)**	**9.7E-03**	**68.1**
*Cell Cycle (R-HSA-1640170)*	*551*	*11*	*85 (15.7%)*	*34 (6.2%)*	*1.0E-02*	*54.1*
*DNA Repair (R-HSA-73894)*	*323*	*12*	*54 (17.1%)*	*17 (5.3%)*	*1.1E-02*	*61.1*
*Organelle biogenesis and maintenance (R-HSA-1852241)*	*310*	*13*	*50 (16.5%)*	*20 (6.5%)*	*2.7E-02*	*66*
Developmental Biology (R-HSA-1266738)	748	14	104 (14.1%)	33 (4.4%)	5.6E-02	57.7
Hemostasis (R-HSA-109582)	693	15	97 (14.2%)	36 (5.2%)	5.6E-02	68
Cell-Cell communication (R-HSA-1500931)	131	16	22 (17.1%)	7 (5.3%)	1.1E-01	68.2
Transport of small molecules (R-HSA-382551)	628	17	75 (12.1%)	28 (4.5%)	4.4E-01	65.3
Neuronal System (R-HSA-112316)	351	18	34 (9.8%)	13 (3.7%)	9.2E-01	47.1
Signal transduction (R-HSA-162582)	2538	19	260 (10.4%)	93 (3.7%)	9.9E-01	58.1

**Bold text**: highly significant (cluster q < 0.01); *Italic text*: significant (cluster q < 0.05); text: cluster not significant, but contains significant (q < 0.05) sub-pathways. The stable identifiers displayed next to the cluster names can be used to retrieve the full pathway information from the Reactome database.

**Table 2:** Pathway analysis of Hepatic DEGs & DEPs detected after 0.1% DMSO exposure.

Cluster name (stable identifier)	Set size	Ranking (DEGs)	Amount DEGs (%)	Amount DEGs (%)|log2FC| > 1	q-value	% DEGs Down-regulated
**Metabolism (R-HSA-1430728)**	**2035**	**1**	**472 (23.7%)**	**272 (13.4%)**	**9.1E-23**	**58.9**
**Vesicle-mediated transport(R-HSA-5653656)**	**619**	**2**	**164 (26.8%)**	**96 (15.5%)**	**4.8E-11**	**51.5**
**Extracellular matrix organization (R-HSA-1474244)**	**295**	**3**	**81 (28.0%)**	**55 (18.6%)**	**2.5E-06**	**77.8**
**Disease (R-HSA-1643685)**	**514**	**4**	**124 (24.5%)**	**77 (15.0%)**	**3.6E-06**	**61.3**
**Immune System (R-HSA-168256)**	**1950**	**5**	**371 (19.5%)**	**220 (11.3%)**	**1.0E-05**	**58.5**
**Muscle contraction (R-HSA-397014)**	**198**	**6**	**58 (29.7%)**	**40 (20.2%)**	**1.4E-05**	**82.8**
**Metabolism of proteins (R-HSA-392499)**	**1506**	**7**	**291 (19.7%)**	**176 (11.7%)**	**9.3E-05**	**59.9**
**Cellular responses to stress (R-HSA-2262752)**	**393**	**8**	**88 (22.7%)**	**59 (15.0%)**	**2.1E-03**	**69.3**
**Hemostasis(R-HSA-109582)**	**693**	**9**	**141 (20.6%)**	**88 (12.7%)**	**3.2E-03**	**56.7**
**Developmental Biology (R-HSA-1266738)**	**748**	**10**	**149 (20.2%)**	**85 (11.4%)**	**4.9E-03**	**69.1**
**Cell-Cell communication (R-HSA-1500931)**	**131**	**11**	**35 (27.1%)**	**21 (16.0%)**	**6.8E-03**	**62.9**
*Programmed Cell Death (R-HSA-5357801)*	*125*	*12*	*33 (26.8%)*	*15 (12.0%)*	*1.0E-02*	*54.5*
Transport of small molecules (R-HSA-382551)	628	13	114 (18.4%)	75 (11.9%)	1.2E-01	58.8
Signal transduction (R-HSA-162582)	2538	14	377 (15.1%)	257 (10.1%)	8.5E-01	64.5
Neuronal system (R-HSA-112316)	351	15	44 (12.6%)	31 (8.8%)	9.8E-01	68.2
Gene expression (R-HSA-74160)	1755	16	239 (14.0%)	145 (8.3%)	1.0E + 0	55.6

**Bold text**: highly significant (cluster q < 0.01); *Italic text*: significant (cluster q < 0.05); text: cluster not significant, but contains significant (q < 0.05) sub-pathways. The stable identifiers displayed next to the cluster names can be used to retrieve the full pathway information from the Reactome database.

Through pathway analysis on DEGs, 225 significantly overrepresented pathways (q-value < 0.05) were found in cardiac MTs, which corresponded to 19 clusters (out of a total of 25 clusters in the Pathway Browser), and 167 pathways corresponding to 16 clusters in hepatic MTs. There was substantial overlap between the tissue types, with 60 pathways and 15 clusters found in both. Although there were differences in magnitude of DMSO effect between tissue types, the affected biological processes by DMSO do not appear to be tissue-specific.

The most significantly affected cluster by DMSO is the “metabolism” cluster in hepatic MTs. Here, most effects were found in pathways “citric acid cycle and respiratory electron transport” (q-value: 3.5 * 10^−10^, 63 DEGs out of 171 genes, 76.2% downregulated), “glucose metabolism” (q-value: 9.9 * 10^−9^, 36 DEGs out of 77 genes, 80.5% downregulated) and “metabolism of lipids and lipoproteins” (q-value: 2.9 * 10^−6^, 165 DEGs out of 728 genes, 55.2% downregulated). Changes in lipid and lipoprotein metabolism were not detected in cardiac MTs using DEGs, but similar effects of DMSO were observed for “citric acid cycle and respiratory electron transport” (q-value: 1.3 * 10^−12^, 58 DEGs out of 171 genes, 65.5% downregulated) and “glucose metabolism” (q-value: 2.8 * 10^−3^, 20 DEGs out of 77 genes, 55.0% downregulated), though less genes were downregulated in cardiac MTs compared to hepatic MTs.

Another highly affected cluster by DMSO treatment in both tissue types was “vesicle-mediated transport”. DMSO effects in this cluster were mainly detected in processes related to Golgi-mediated protein transport and secretion. Of this process, “ER-to-Golgi anterograde transport” was the most affected (Cardiac: q-value: 2.4 * 10^−6^, 38 DEGs out of 134 genes, 68.4% downregulated; Hepatic: q-value:1.0 * 10^−5^, 44 DEGs out of 134 genes, 44.4% downregulated). This pathway was also part of the cluster “metabolism of proteins”, which additionally revealed DMSO effects on “Asparagine N-linked glycosylation” (Cardiac: q-value: 2.1 * 10^−6^, 64 DEGs out of 283 genes, 76.6% downregulated; Hepatic: q-value: 1.2 * 10^−7^, 83 DEGs out of 283 genes, 54.8% downregulated), which were post-transcriptional protein modifications necessary for transport of proteins from the ER to the Golgi^17^.

Though the cluster of “cellular responses to stress” was detected in both tissue types, overrepresented pathways differ. “Cellular senescence” was significantly affected in cardiac MTs (q-value: 2.6 * 10^−4^, pathway size: 192, 42 DEGs, 64.3% downregulated), but not detected during pathway analysis in hepatic MTs. Finally, additional cardiac-specific clusters affected by DMSO were “cell cycle”, “DNA repair”, “organelle biogenesis and maintenance”, but also the highly significant cluster (q < 0.01) of “chromatin organization”. Since the most significantly affected pathways were found in both tissue types, this indicates robust actions of DMSO.

### DMSO effects observed in regulation of gene expression and translation

Pathways related to regulation of gene expression and translation were already detected during the pathway analysis of cardiac MTs (Table 3). Though these pathways were not overrepresented in hepatic MTs, the amounts of DEGs detected indicated that DMSO was able to influence these processes and could induce biological alterations to the cell model.

**Table 3:** Pathways related to transcriptional regulation.

	Set size	Cardiac			Hepatic		
		DEGs	q-value	% DEGs down-regulated	DEGs	q-value	% DEGs down-regulated
Gene silencing by RNAs	134	35 (26.7%)	3.8E-05	74.3	25 (19.1%)	3.6E-01	72.0
Transcriptional regulation by small RNAs	108	32 (30.2%)	7.6E-06	75.0	24 (22.6%)	1.4E-01	75.0
MicroRNA biogenesis	13	5 (41.7%)	4.0E-02	60.0	1 (8.3%)	9.6E-01	0.0
Epigenetic regulation of gene expression	154	31 (20.5%)	6.8E-03	61.3	27 (17.9%)	4.5E-01	77.7
DNA methylation	68	18 (27.3%)	3.4E-03	66.6	13 (19.7%)	4.4E-01	92.3

#### DMSO effects observed in regulation by microRNAs

Tissue-specific influence of DMSO was observed in the sequencing data of miRNAs. Out of 1,105 sequenced cardiac miRNAs, 704 (=63.7%) were differentially expressed (DE, FDR <0.05) with 59.5% showing downregulation. Furthermore, the PCA plot (Fig. 2c) revealed a clear difference between the miRNAs of the treatment groups. In hepatic MTs, out of 1,033 sequenced miRNAs, 186 (=18%) were DE with approximately half of the miRNAs being upregulated (47.3%). Furthermore, the PCA plot not only revealed a clear separation between miRNAs of the treatment groups, but indicated also a time-dependent effect. The two principal components (time and dose) represented together more than 95% of the total variation, leaving only minor effect for other factors.

**Figure 3:**
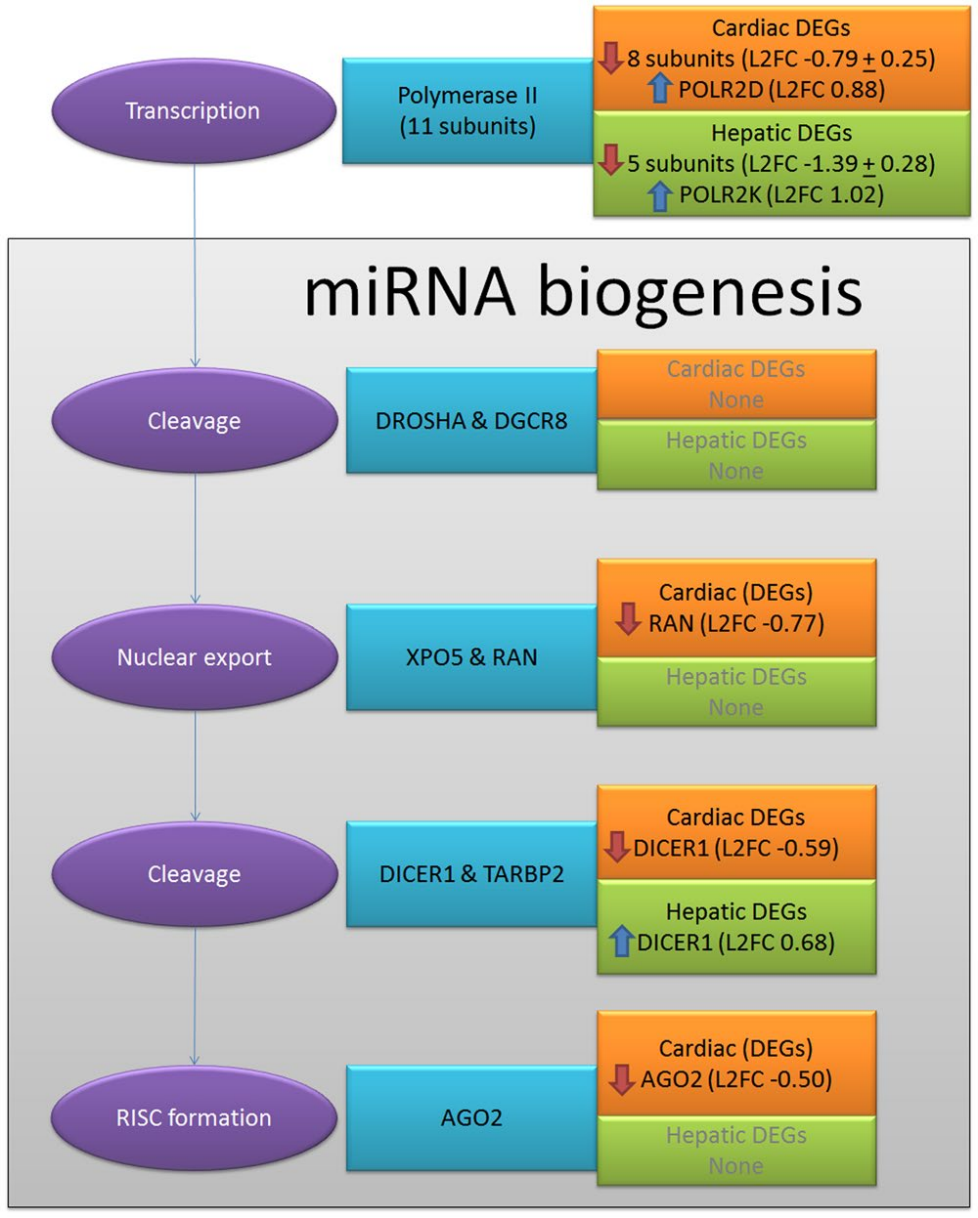
DMSO effect in the process of Gene silencing by RNAs. DEGs in biogenesis of miRNA. The complete process is divided in sub-processes (purple ovals) and depicting involved genes (blue rectangles) and detected DEGs for cardiac (orange rectangles) and hepatic (green rectangles) samples.

To investigate the source of tissue-specific difference, gene expression changes in the process of miRNA biogenesis were investigated in more detail (Fig. 3). MiRNA biogenesis starts by the transcription of the primary miRNA transcribed by polymerase II (a complex of 11 subunits) or polymerase III (a complex of 10 subunits). In cardiac MTs, polymerase II contained 1 upregulated and 8 downregulated genes. In hepatic MTs, 5 genes were downregulated and 1 was upregulated. Though fewer genes were downregulated in hepatic MTs, the fold changes were larger compared to cardiac MTs. The cleavage of the pri-miRNA into pre-miRNA did not appear affected by DMSO exposure. *DICER1* (which cleaves pre-miRNAs) was downregulated in cardiac MTs and upregulated in hepatic MTs, depicting a clear tissue-specific difference in the response to DMSO exposure. Finally, *AGO2* (encoding the main component of the miRNA-RISC) was downregulated in cardiac MTs. The changes in this process could induce differences in the cells miRNA content and therefore affect their regulatory function.

In cardiac MTs, the downregulated genes in miRNA biogenesis could explain the extremely high amount of downregulated DE miRNA and indicate extreme deregulation of gene silencing by miRNAs. In hepatic MTs, where the only significant change was the upregulation of *DICER1* (Table 3), miRNA biogenesis was not disrupted by DMSO exposure.

To get an indication of the DMSO effects on gene silencing by miRNAs, the miRTarBase database^18^, containing experimentally validated microRNA-target interactions (MTI), was used to obtain gene targets of detected DE miRNAs. We only included MTIs with strong evidence (validated by reporter assay, Western blot or qPCR). Of the 704 DE cardiac miRNAs, only 281 (=40%) could be found in the database, resulting in a total of 2051 gene targets potentially affected by DMSO-induced changes in miRNA gene silencing. For hepatic MTs, targets for 106 DE miRNAs (=57%) were obtained, with a total of 545 potentially affected genes. The obtained gene targets were used to visualize overrepresented pathways using the Reactome Pathway Browser (Supplementary Fig. 3). Unexpectedly, overrepresented pathways were located in the same clusters for both tissue types. Most effects were observed for “Signal Transduction”, “Immune System” and “Gene Expression”. However, extreme deregulation of cardiac MTs and limited information about MTIs is making any downstream analysis on putative affected mRNA irrelevant.

#### DMSO effects observed in epigenetics

**Figure 4:**
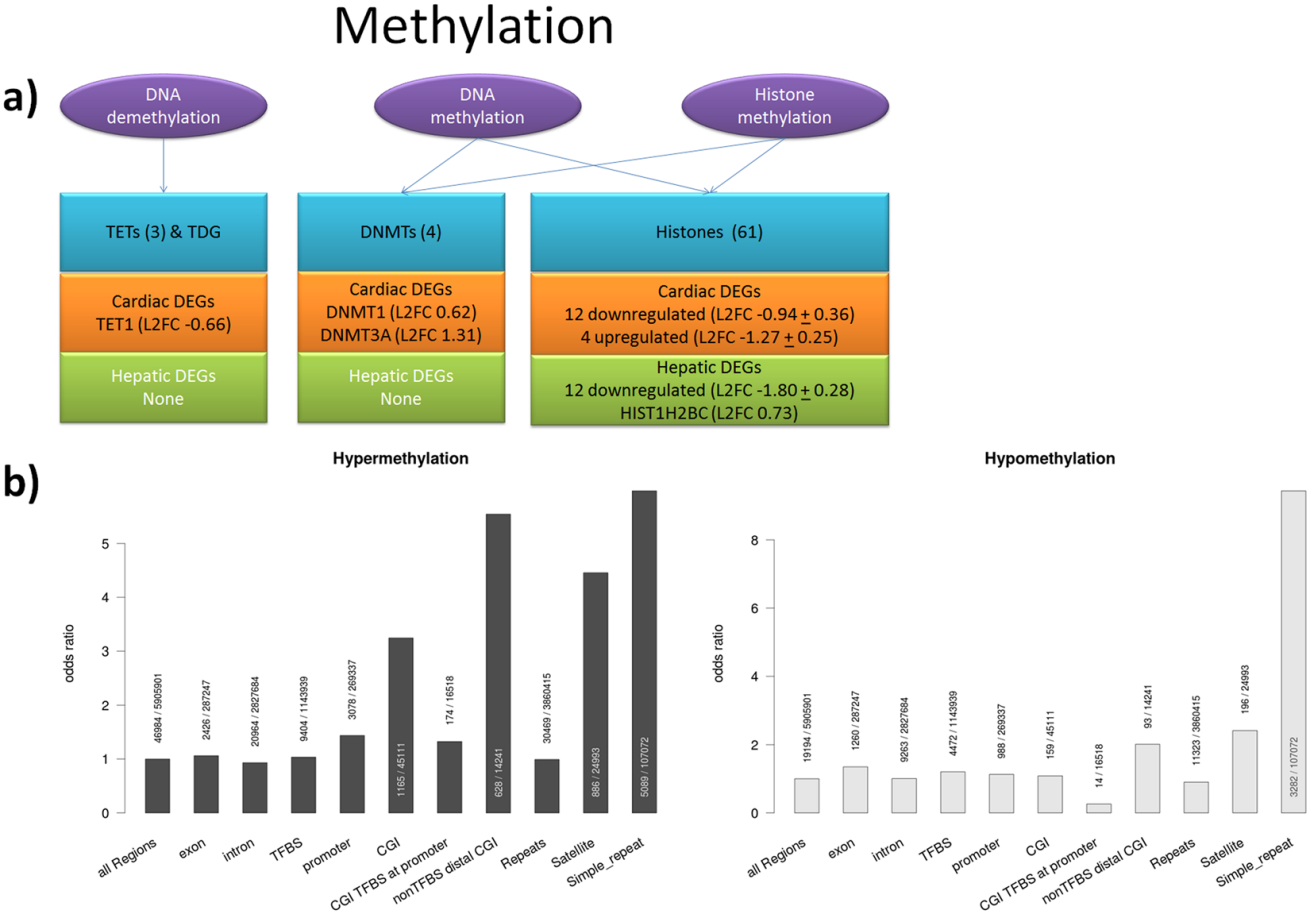
Epigenetic regulation of gene expression. (**a**) DEGs involved in DNA methylation. The process is divided in sub-processes (purple ovals) and depicting involved genes (blue rectangles) and detected DEGs for cardiac (orange rectangles) and hepatic (green rectangles) samples. (**b**) Relative enrichment of DMRs (DMSO vs UNTR, FDR 5%) within genomic features. For each feature, the number of overlapping DMRs over the total number of tested windows for the respective feature is depicted. Compared to all genomic regions, hypermethylated regions are enriched for satellites, simple repeats, and CGIs distal to promoters without known regulatory evidence and hypomethylated regions are enriched for simple repeats.

In order to assess epigenetic alterations introduced by DMSO, we focus on genome wide DNA methylation. Pathway analysis of cardiac gene expression revealed deregulation of DNA methylation pathways. Methyltransferases *DNMT1*, a key factor for maintenance of DNA methylation, and *DNMT3A*, facilitating both *de novo* and maintenance of DNA methylation, were upregulated while *TET1*, which plays a key role in active de-methylation, was downregulated in cardiac MTs (Fig. 4a). Upregulation of epigenetic writers and downregulation of erasers after DMSO treatment pointed towards genomic hypermethylation, which potentially reduced transcriptional activity. In contrast, in hepatic pathway analysis, deregulation of DNA methylation was not observed. Note that, in both tissue types, transcriptional evidence for deregulation of other related epigenetic mechanisms were observed, such as histone methylation, where 16 genes and 13 genes were differentially expressed in cardiac and hepatic MTs respectively.

Whole genome methylation profiling by MeDIP-seq revealed 66,178 differentially methylated regions (DMRs; q-value < 0.05) in cardiac MTs. These alterations affected 1.1% of the covered genome. In line with the observed transcriptional changes of writers and erasers of DNA methylation, 71% of the DMRs corresponded to gain of methylation (46,984 hypermethylated regions vs 19,194 hypomethylated regions). In contrast, in hepatic MTs, no DMRs passed correction for multiple testing. Furthermore, the PCA plot (Fig. 2d) indicated a difference between treatment groups in cardiac MTs but not in hepatic MTs. Together, this illustrated tissue-specific impact of DMSO on the methylome in maturing cardiac MTs, while the mature hepatic MTs appeared unaffected.

In order to analyze the regulatory effects of the DMRs, the regions were annotated with known regulatory features, such as promoters, CpG Islands (CGIs), transcription factor binding sites (TFBS), and different classes of repetitive elements (Fig. 4b). Both hypo- and hypermethylated regions enriched specific repeat classes such as satellites (odds ratio of hyper/hypomethylation: 4.4/2.4) and simple repeats (hyper/hypomethylation 6.0/9.4 odds ratio), while repetitive elements in general were not enriched. The effect of DMSO on the upregulation of DNMTs and subsequent hypermethylation of repeat sequences was in line with previous findings in mouse embryoid bodies^19^. As expected, hypermethylated regions were also highly enriched in CGIs without regulatory evidence, e.g. not overlapping with TFBS and distal to transcription start sites. The odds ratio of these regions over the genome was 5.5. While CGIs in general had an odds ratio of 3.2, there was no enrichment of CGIs overlapping known TFBS at promoters, the regions with the best studied regulatory potential. Just as promoter regions, other features such as exons, introns, and TFBS were affected by hypermethylation at approximately the odds of the captured genomic background (Fig. 4b). Although we found specific examples where promoter hypermethylation co-occurs with transcriptional repression, no global correlation between differential promoter methylation and gene expression was observed. Only 148 of the 1,436 genes (10.3%) featuring hypermethylated promoters were downregulated in DMSO treated cardiac MTs. 86 of the genes (6%) were overexpressed, contrary to the expected repressive effect of methylation.

Taken together, our data implied a fundamental influence of DMSO on DNA methylation in cardiac MTs, but not in hepatic MTs. The induced alterations affected preferentially satellites and simple repeats, as well as CGIs distal to promoters without known regulatory evidence. The expression of neighboring genes was not significantly correlated to methylation differences. We therefore conclude that transcriptional repression by promoter hypermethylation was not the primary regulatory effect of the altered methylation marks, but the findings indicate a global disruption of DNA methylation mechanisms.

## Discussion

While high doses of DMSO were initially investigated for medical applications, lower doses are now commonly used as solvent for toxicology or for cryopreservation of cell cultures, oocytes and embryos. In the meantime, biological effects of DMSO have been forgotten or considered negligible. With the evolution of biomedical science towards more sensitive high-throughput techniques and towards new areas of research, including epigenome modifications and microRNA-mediated gene silencing, we evaluated the biological effects of DMSO exposure on the transcriptome and the epigenome.

The first overview of DMSO-induced cross-omics effects, observed through amounts of differentially changed entities and PCA analysis, indicated an increased susceptibility to DMSO effects in cardiac MTs. Differences between the investigated tissue types may relate to the fact that the cardiac model contains iPSC-derived human cardiomyocytes that are still maturing, while the hepatic model contains mature human hepatocytes. Currently, it is not yet possible for iPSC cells to reach an adult-like phenotype^20,21^ and the concise review on iPSC-derived human cardiomyocytes of Robertson *et al*.^22^ describes iPSCs to better resemble fetal cardiomyocytes (regarding structure, proliferation, metabolism and electrophysiology) rather than adult cardiomyocytes. Our observations of DMSO effects, notably the extreme epigenetics modifications in fetal-like cardiac cells, are particularly relevant for cryopreservation during assisted reproductive technology (embryos, oocytes and sperm cells). Another difference to be considered between our two cell models is the number of donors. Indeed, while the cardiac MTs are composed of IPSC-derived cardiomyocytes from a single donor (co-cultured with fibroblasts also from 1 donor), hepatic MTs contain mature hepatocytes from 10 donors (co-cultured with Kupffer cells of 1 donor). While a single-donor model is more comparable to an *in vivo* situation then a multi-donor model, single-donor cardiac MTs might be more susceptible for bias due to genotype sensitivity than our multi-donor hepatic MTs that have the ability to even out these effects.

DMSO effects on cellular processes were analyzed using full transcriptome data. 2,051 DEGs were identified for cardiac MTs and 2711 DEGs for hepatic MTs. In both tissue types, downregulations are found for 60% of DEGs. Pathway overrepresentation analysis using DEGs, highlights similar DMSO effects in both tissue types. This indicates robust actions of DMSO that may possibly be extrapolated to other tissues. Observed changes in cellular processes include changes in mitochondrial pathways linked to ROS production and cellular ATP generation. DMSO is a known radical scavenger^23^. While this property of DMSO may provide protection against high levels of ROS, at normal or decreased levels of ROS, it may hamper basal cell metabolism by scavenging electrons needed for ATP production^24^. The resulting decrease in ATP content was observed in our two DMSO treated cell models (Supplementary Fig. 2). Cardiac MTs showed a steep decrease over time (87% decrease after 2 weeks of exposure), while the ATP content of hepatic MTs show a small decrease initially (36% decrease in the first 72 h), after which ATP content slowly recovered to baseline level. Different downstream effects are relevant depending on the biological application of DMSO. For instance, for cell assays and toxicological research, changes in the amount of ROS or free energy in the cell impact on the capability of the cell to deal with induced stresses, thereby potentially leading to erroneous interpretation of results from *in vitro* assays. Within assisted reproductive technology, the ATP content is a good predictor of embryo viability. The DMSO-induced ATP decrease, especially in the cleavage-stage, can induce downstream effects that may disrupt cellular function, implantation ability and fetal development^25,26^. DMSO-induced reduction in implantation rates and pregnancy losses were already observed from animal models^27^.

Next to overlap in DMSO-affected processes between the tissue types, pathway analysis also revealed tissue-specific differences. The higher amount of affected processes detected in cardiac MTs may indicate increased susceptibility of cardiac MTs to the effects of DMSO, though there might also be a statistical bias due to different amount of DEGs used in the pathway analysis. The most significant difference is the detection of “chromatin organization” in cardiac MTs, while this cluster is not observed in hepatic MTs. Cardiac MTs also show more effects in other processes that regulate gene expression. Results of miRNA analysis revealed complete deregulation of cardiac miRNA biogenesis and miRNA content. Though changes in hepatic miRNA biogenesis were minimal, still 18% of detected miRNAs were significantly changed by DMSO exposure. Genome-wide methylation effects upon DMSO treatment show large tissue-specific variation. While there is no significant difference with respect to hepatic MTs, strong effects are observed with cardiac MTs, in particular with respect to specific repetitive sequences such as satellites and simple repeats. DNA repetitive elements account for 50% of the human genome and methylation in these regions suppresses their mobility and maintains genomic stability. Changes in the methylation status of repeats after DMSO treatment might destabilize the genome, implying phenotypic and pathological consequences. Indeed, recently, methylation changes of repetitive sequences, in particular satellite repeat elements, have been found in cardiomyopathic heart tissue compared to normal heart tissue^28^. Furthermore, it has been shown that DMSO changes genome-wide DNA methylation profiles dependent on specific gene loci. This may be due to drastic stimulation of *DNMT1* and *DNMT3*. We observed upregulation of *DNMTs*, but literature also reported increased protein levels and catalytic activity by interaction of DMSO with their enzymes substrates (DNA and AdoMet)^19,29,30^. Furthermore, it is also possible that DMSO acts as a methyl donor to induce hypermethylation^31^. However, it has to be kept in mind that there is an interplay between DNA methylation and chromatin configuration, which is regulated by more processes than just DNMTs. Overall, we speculate the effects of DMSO on methylation changes to be a global deregulation characterized by a genome wide hypermethylation. Cell mechanisms in charge of removing detrimental methylations at important regions, such as TFBS, may then have removed the methylation changes with a negative outcome on cell survival, leaving an over-representation of DMR in the repetitive elements. This is in analogy to mutation rates, which are higher for repetitive elements since they are less subjected to natural selection and DNA repair mechanisms^32^. We believe that the methylation difference between mature hepatic and maturing cardiac cells may be detrimental for cryopreserved cells and especially for oocytes and embryos. Previously reported DMSO effects on oocytes and embryos are limited to changes in gene expression in animal models^27,33–36^. We now showed, for the first time, the influence of DMSO on the epigenome of human cells *in vitro*. It is uncertain if exposed cells can recover from temporary DMSO exposures. Increased survival has been documented for embryos after withdrawal from 2% DMSO for less than 24 h, though this was not the case with exposures longer then 48 h^27^. While methylation changes have an adaptive nature, they may also be persistent. From research conducted on the Dutch famine cohort, it is known that the periconceptional period (before conception until early pregnancy) is crucial for establishing and maintaining epigenetic marks. During this period epigenetic marks present on the parental DNA are removed to produce a totipotent zygote which is reprogrammed after implantation^37^. Exposures during this time may induce persistent epigenetic differences, which can result in disease later in life or possibly be inherited by the offspring to induce transgenerational effects^38–42^. It is already well known that infants conceived through assisted reproductive technologies are prone to be born preterm, have low birth weight and even have a significantly increased risk of birth defects^43,44^. Furthermore, some short-term health outcomes were slightly poorer in children conceived by IVF, though overall the outcomes were positive^42,45^. Regarding long-term health and development, though available data is limited, cardiovascular and metabolic risk factors that may ultimately result in chronic cardiometabolic disease have been indicated^42^. Furthermore, also epigenetic risks have been debated, mostly focusing on methylation and imprinting. For the most studied imprinting disorders Beck-with-Wiedemann syndrome and Angelman syndrome, the incidence was higher for children born after assisted reproductive technologies compared to natural conception^41,46–50^. While there are many factors contributing to these abnormalities, (decreased infertility, higher age of the parents, technical interference with a biological process, etc.) DMSO-induced effects may be one of them.

In the last decade, improvements made in artificial reproductive technology greatly increased the success rate of IVF treatments. Especially, improved cryopreservation techniques (transition to vitrification) aided greatly. Though not proven yet, increased success rates are reported when implanting previously frozen embryos^51–53^. This induced the “freeze all” trend in the clinic, which greatly increases the need for understanding consequences of cryopreservation at the molecular level. Furthermore, while storing frozen oocytes was initially intended for women threatened by decreased fertility due to disease, in our carrier-focused society, it has also become a method to prevent age-related infertility for women who want to delay motherhood^54^. The most recent development in assisted reproductive technology is the emerging of concerns about DMSO effects. While there is still controversy on this topic, the field is moving towards DMSO-free methods. However, DMSO-free does not automatically mean safe because of the use of other cryoprotectants^55–57^.

## Conclusions

Our study highlights the capability of DMSO to induce changes in cellular processes in both cardiac and hepatic cells, but more severely, induce alterations in miRNA and epigenetic landscape in the 3D maturing cardiac model. The changes in cellular processes can have consequences for conclusions drawn from cell assays and therefore also in any application of these findings (e.g. false negative drug toxicity conclusions). Furthermore, the extreme changes in miRNA and alterations in the epigenetic landscape may pose a threat, especially for assisted reproductive technology. Genome-wide hypermethylation induced by global deregulation of methylation mechanisms, especially when it affects genes important in development, may have negative consequences directly, later in life or possibly in later generations.

Overall, use of DMSO should be avoided where possible. However, for the time being, DMSO is indispensable within biotechnological applications. In these cases, the effects that DMSO may have should be considered and the concentration should be kept as low as possible, because even at low concentrations DMSO is not inert.

## Methods

### Samples

In this study, 3D InSight^TM^ Human Cardiac Microtissues (InSphero, for beta-testers) were used, containing a co-culture of approximately 4000 iPSC-derived human cardiomyocytes from a female Caucasian donor with no known disease phenotype and 1000 cardiac fibroblasts from an 18 year old Caucasian male. The MTs were cultured in 3D Insight^TM^ Human Cardiac Microtissue Maintenance Medium (InSphero). Furthermore, 3D InSight^TM^ Human Liver Microtissues (InSphero) were used, approximately 1000 primary human hepatocytes (multi-donor pool of 5 males and 5 females between 7–59 years old) in co-culture with 1000 primary human Kupffer cells from a Caucasian 27 year old of unreported gender. The MTs were cultured in 3D Insight^TM^ Human Liver Microtissue Maintenance Medium- AF (InSphero).

### Exposure

The microtissues (MTs) were exposed to medium only (=untreated) or medium containing 0.1% DMSO for two weeks. Medium was changed three times daily, in concordance with the experimental design of our current research^58^. During the 2 week exposure, there were a total of 7 sampling time points at 2, 8, 24, 72, 168, 240 and 336 hours, which were sampled in triplicate (total 21 samples per condition). Per sample, 36 cardiac MTs or 54 hepatic MTs were used in order to obtain sufficient amounts of RNA for sequencing.

### RNA sequencing sample preparation

Total RNA was isolated using the AllPrep DNA/RNA/miRNA Universal Kit (Qiagen). The sample was depleted of ribosomal RNA using the Ribo-Zero Gold rRNA Removal kit (Human/Mouse/Rat) (Illumina®) and prepared for sequencing with the SENSE total RNA library preparation kit (Lexogen). After library preparation, the quality was assessed on Agilent 4200 TapeStation and library concentration was determined by Qubit^TM^ before sequencing (paired-end 100 bp) on the HiSeq2000. Two files per sample were obtained, split in left and right reads, and Lexogen adapter sequences (first 12 bases of all reads) were removed using Trimmomatic^59^ (v.0.33). The quality of the sequencing data was checked using FastQC^60^ (v.0.10.1) before and after trimming. For cardiac samples, no samples were discarded because of poor quality. Two samples were discarded because of low read counts (UNTR_002_3 and UNTR_240_3) and principal component analysis (Supplementary Fig. 1) revealed 4 outliers which were removed (UNTR_008_3, UNTR_168_2, UNTR_240_2 and DMSO_336_2). Finally, because only one replicate of 240 hour untreated samples remained, all samples of this time point were removed from statistical analysis. For hepatic samples, no samples were discarded.

### RNA data-analysis

Read counts for genes were obtained by aligning the reads to the reference genome (Genome Reference Consortium Human Build 38, GRCh38.p7) using RSEM^61^ (v.1.2.28) with the paired-end and Bowtie2^62^ (v.2.2.6) option. Thereafter, the read counts for each gene were used for determination of differentially expressed genes using the DESeq2^63^ R package (v.1.16.1). Here, the design was set according to exposure (UNTR or DMSO), other settings were kept to their default parameters, except for minimum count, which was set to an average read count of one across all samples. Finally, remaining ribosomal genes were filtered out of the dataset to ensure complete ribosomal depletion of the data.

### MiRNA sequencing sample preparation

An aliquot of the isolated total RNA was selected on size and ligated using the TruSeq Small RNA Library Prep Kit (Illumina®). After library preparation, the samples were sequenced (paired-end 100 bp) on the HiSeq2000. Adapter sequences were trimmed^64^ and reads between 16 and 35 bp were kept for analysis. Three samples were discarded because of low read counts (Cardiac: UNTR_168_1, UNTR_168_2 and Hepatic: DMSO_002_3). Furthermore, principal component analysis (Supplementary Fig. 1) revealed no outliers.

### MiRNA data-analysis

MiRNA data analysis was done similarly as described previously^65^, except for the use of mirDeep2, which does not gain additional information when using human samples. In short, PatMaN^66^ (v.1.2.2) was used to align trimmed reads to the human genome without allowing mismatches or gaps. To obtain complete read counts for 3’ and 5’ miRNA species, the mapping output was parsed.

### Proteomics

Proteomics data was also obtained and analyzed. Methods and results are included in Supplementary data.

### MeDIP sequencing

For preparation of MeDIP-Seq libraries, the low input MeDIP protocol^67^ was modified. DNA was fragmented to 100–200 bp using the Covaris S2 system. Because of lower DNA yield for cardiac 0.1% DMSO samples, the triplicates were pooled before fragmentation. End repair and A tailing was performed using the NEBNext® Ultra^TM^ library prep kit for Illumina® (NEB), adapters were ligated with NEBNext® Ultra^TM^ Ligation Module (NEB) and samples were purified using Agencourt® AMPure® XP beads (Beckman Coulter). Methylated fragments were captured using the MagMeDIP kit (Diagenode). In short, denatured DNA was mixed with anti-5-meC-antibody and captured using magnetic beads. Capture efficiency was determined by qPCR against spiked-in Lambda-DNA fragments in precapture and postcapture library samples. Libraries were amplified in a final PCR step using barcoded TruSeq primers. Quality was assessed on Agilent Bioanalyzer 2100 and library concentration was determined by Qubit^TM^ and qPCR.

### MeDIP data-analysis

In order to gain exhaustive genome-wide coverage the triplicate samples that have been sequenced individually were merged before alignment. MeDIP sequencing reads were aligned to the GRCh38 reference genome using bwa Version 0.7.15-r1140^68^, and analyzed in 250 base windows using the R/bioconductor package QSEA^69^ (v.1.4.0) with standard parameters. Within QSEA, the MeDIP enrichment was calibrated with 450k methylation array measurements of primary hepatocytes (GSM999339) and cardiac myocytes (HCM, GSM999381) from ENCODE^70^, for the hepatic and cardiac micro tissues respectively. To this end, beta values of the calibration samples were computed with the R/bioconductor package Minfi^71^ (v.1.24.0), genomic locations of the array probes were mapped from GRCh37 to GRCh38 using the UCSC liftOver command line tool^72^, and probes within 250 base windows were averaged. Differentially methylated regions obtained from QSEA were annotated with gene, exon, and promoter (transcription start site +/− 2 kilobases) information from RefSeq, ENCODE TFBS and model based CpG islands, all obtained via the UCSC table browser. Since ENCODE TFBS were not available for GRCh38, genomic locations were mapped from GRCh37 using the liftOver tool.

## Supplementary information

Supplementary Information

## Data Availability

The datasets generated and analyzed during the current study are available in the BioStudies database (http://www.ebi.ac.uk/biostudies) under accession numbers S-HECA1, S-HECA5, S-HECA9, S-HECA18, S-HECA33, S-HECA34, S-HECA47, S-HECA139, S-HECA158, S-HECA361, S-HECA401, S-HECA403.
