# Supplementary material for: DMSO induces drastic changes in human cellular processes and epigenetic landscape in vitro
Source: Sci Rep. 2019 Mar 15;9:4641. doi: 10.1038/s41598-019-40660-0 (PMC6420634; doi:10.1038/s41598-019-40660-0)
Supplement: Supplementary file 1 — Supplementary Information [file 41598_2019_40660_MOESM1_ESM.pdf]

DMSO induces drastic changes in human cellular processes and epigenetic landscape *in vitro*

M. Verheijen, M. Lienhard, Y. Schrooders, O. Clayton, R. Nudischer, S. Boerno, B. Timmermann, N. Selevsek, R. Schlapbach, H. Gmuender, S. Gotta, J. Geraedts, R. Herwig, J. Kleinjans, and F. Caiment*.

Supplementary Information

Content:

Supplementary data

Supplementary data: Proteomics Methods & Results

Supplementary figures

Supplementary figure 1: PCA plot to detect outliers.
Supplementary figure 2: Changes in microtissue ATP content over time.
Supplementary figure 3: Indication of biological effects due to miRNA changes.

Supplementary tables

Supplementary table 1: Hierarchically ordered overrepresented pathways (using DEGs) in cardiac MTs after 0.1% DMSO exposure
Supplementary table 2: Hierarchically ordered overrepresented pathways (using DEGs) in hepatic MTs after 0.1% DMSO exposure
Supplementary table 3: Hierarchically ordered overrepresented pathways (using DEPs) in cardiac MTs after 0.1% DMSO exposure
Supplementary table 4: Hierarchically ordered overrepresented pathways (using DEPs) in hepatic MTs after 0.1% DMSO exposure

Supplementary data: Proteomics Methods & Results

Proteomics methods

Proteomics sample preparation

Proteins were isolated from pooled MTs by resuspending in 100 µl lysis buffer containing 8M Urea, 1 mM Dithiothreitol, 0.1M Ammonium bicarbonate, pH 7.8. After four freeze-thaw cycles, the samples were centrifuged at 16000xg for 15 min at 4° C and protein concentrations were assessed with the Qubit™ Protein Assay Kit (Invitrogen, Molecular Probes). Protein isolates were then reduced with 12 mM dithiothreitol for 30 min and 37°C and alkylated with 40 mM iodoacetamide for 30 min in the dark before diluting the samples with 0.1 M ammonium bicarbonate to a final concentration below 2M. Proteins were digested by incubation with trypsin (Promega) to a final enzyme:subtrate ratio of 1:100. Digestion was stopped by adding formic acid to a final concentration of 1%. The peptides were cleaned up using Sep-Pak tC18 cartridges (Waters) according to the manufacturer's instructions, and eluted with 60% ACN and 0.1% formic acid (Sigma-Aldrich). The resulting peptides were evaporated to dryness on a vacuum centrifuge and stored by -80°C.

Proteomics mass spectrometry measurements

Peptides were measured on a Orbitrap Fusion mass spectrometer (Thermo Fisher Scientific) coupled to a NanoLC-2D HPLC system (Eksigent). Samples were loaded onto a self-made column (75 µm × 150 mm) packed with reverse-phase C18 material (ReproSil-Pur 120 C18-AQ, 1.9 µm, Dr. Maisch HPLC GmbH). Peptides were loaded on the column from a cooled (4°C) Eksigent autosampler and separated with a linear gradient of acetonitrile/water, containing 0.1 % formic acid, at a flow rate of 300 nl/min. A gradient from 5 to 30% acetonitrile in 60 minutes was used. The mass spectrometer was set to acquire full-scan MS spectra (300–1500 *m/z*) at 120,000 resolution at 200 *m/z*; precursor automated gain control (AGC) target was set to 500,000. Charge-state screening was enabled, and precursors with +2 to +7 charge states and intensities >5,000 were selected for tandem mass spectrometry (MS/MS). Ions were isolated by use of the quadrupole mass filter with a 1.6 *m/z* isolation window. Wide quadrupole isolation was used, and injection time was set to 50 ms. The AGC values for MS/MS analysis were set to 2,000 and the maximum injection time was 300 ms. HCD fragmentations were performed at a normalized collision energy (NCE) of 35%. MS/MS were detected in the ion trap in

centroid mode. Precursor masses previously selected for MS/MS measurement were excluded from further selection for 25 s, and the exclusion window was set at 10 ppm.

Proteomics data-analysis

Raw MS data were processed using Genedata Expressionist software (v.11.0), consisting of two modules: Refiner MS (data pre-processing) and Analyst (data post-processing and statistical analysis). In short, after noise reduction and normalization, LC-MS peaks were detected and their properties calculated (m/z and RT boundaries, m/z and RT center values, intensity). Individual peaks where grouped into clusters and MS/MS data associated to these clusters were annotated with MS/MS Ions Search (Mascot 2.6) using Peptide Tolerance: 10.0 ppm, MS/MS Tolerance: 0.50 Da, Max Missed Cleavages: 2 and database: Uniprot Swiss-Prot 29062016, Taxonomy *Homo sapiens* (human). Results are validated by applying a threshold of 5% normalized False Discovery Rate (FDR). Protein interference was done based on peptide and protein annotations. Redundant proteins were ignored according to the Occam's razor principle, and at least 2 peptides were required for a positive protein identification (shared peptides were ignored). Protein intensities were computed using the Hi3 method. A maximum of the top 3 peptides per protein (based on the average intensity across samples) was used in the calculation. If a peptide was identified in multiple charges (2+, 3+, 4+) and modification states (Carbamidomethyl (C), Deamidated (NQ) or Oxidation (M)), values were consolidated into a single peptide intensity. For cardiac, 1,652 proteins were found for untreated MT and 1,561 for 0.1% DMSO exposed MT. For hepatic, untreated and DMSO exposed MT resulted in 1593 and 1514 proteins resp. From the cardiac MT untreated group the sample 20160303_021_412, T168 replicate 1, was excluded because it appeared as a clear outlier. After the data pre-processing, the intensities were log2 transformed. The transformed data of each sample were shifted to the median of the medians determined by reference groups consisting of 1,284 proteins, found in all cardiac MT samples (untreated and DMSO treated) and of 1,293 proteins found in all hepatic MT samples. Finally, 2-sided T-tests were used for the determination of differentially expressed proteins (DEPs).

Proteomics results

Protein analysis resulted in 1041 differentially expressed proteins (DEPs; FDR <0.05) in cardiac MTs and 650 DEPs in hepatic MTs, of which 46.3% and 52.3% were downregulated respectively. Figure P1 reveals a clear separation between UNTR and 0.1% DMSO indicated that DMSO was able to affect cellular processes by altering cellular protein levels.

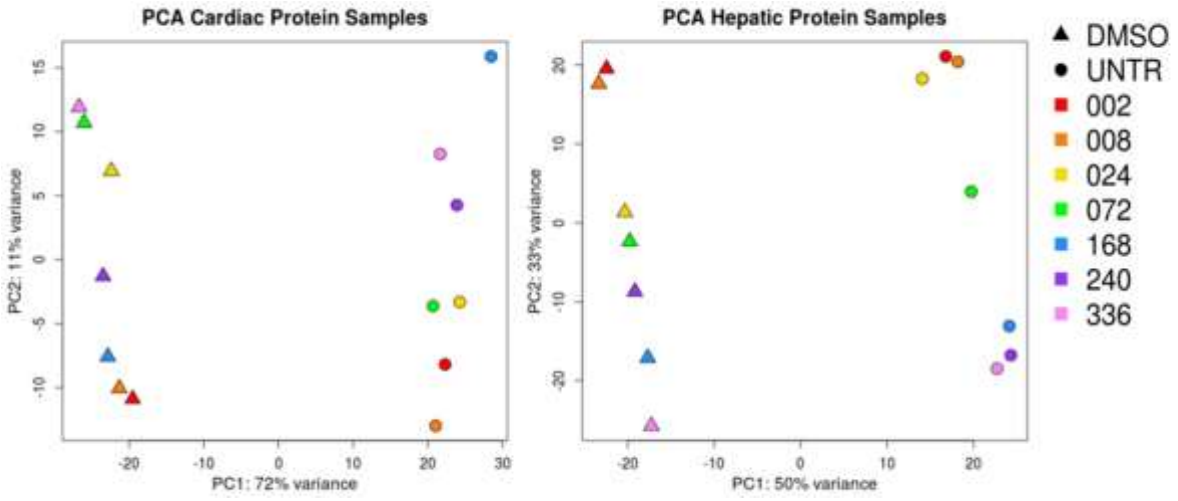

Figure P1: PCA of proteins showing extreme differences in protein content between DMSO (triangle) and UNTR (circles) for both tissue types (cardiac on the left and hepatic on the right).

Table P1 for cardiac and Table P2 for hepatic MTs contain the amount of DEPs and the percentage of downregulated proteins for each cluster, obtained through pathway analysis performed on DEPs (supplementary tables 3&4). Tables P1 & P2 reveal not only differences between tissue types were observed, but also between omics platforms. The transcriptome only indicated changes in “detoxification of reactive oxygen species” (q-value: $3.9*10^{-2}$, 12 DEGs out of 36 genes, 66.7% downregulated) for hepatic MTs, while the proteome identified more DEPs in cardiac (15 DEPs, 53.4% downregulated) as compared to hepatic (6 DEPs, 50.0% downregulated). This phenomenon was also found for the “apoptosis” pathway (Hepatic transcriptome: q-value: $7.33*10^{-3}$, pathway size: 122, 33 DEGs, 54.5%; Hepatic proteome: 18 DEPs, 72.3% downregulated; Cardiac proteome: 27 DEPs, 30% downregulated) in the cluster of “programmed cell death”.

Table P1: Pathway analysis of Cardiac DEGs & DEPs detected after 0.1% DMSO exposure

Cluster name (stable identifier)	Set size	Ranking (DEGs)	Amount DEGs (%)	Amount DEGs (%)|log2FC|>1	q-value	% DEGs Down-regulated	Amount DEPs (%)	% DEPs Down-regulated
**Cellular responses to stress** (R-HSA-2262752)	393	**1**	**91 (23.5%)**	**28 (7.1%)**	**2.2E-09**	**61.5**	**70 (18.1%)**	**28.6**
**Disease** (R-HSA-1643685)	514	**2**	**109 (21.5%)**	**33 (6.4%)**	**4.5E-09**	**61.5**	**58 (11.6%)**	**50.0**
**Vesicle-mediated transport** (R-HSA-5653656)	619	**3**	**122 (20.0%)**	**48 (7.8%)**	**2.7E-08**	**55.7**	**82 (13.4%)**	**56.1**
**Metabolism of proteins** (R-HSA-392499)	1506	**4**	**236 (16.0%)**	**80 (5.3%)**	**9.3E-07**	**68.2**	**259 (17.6%)**	**42.1**
**Chromatin organization** (R-HSA-4839726)	274	**5**	**58 (21.3%)**	**19 (6.9%)**	**5.2E-05**	**53.4**	**34 (12.6%)**	**5.9**
**Muscle contraction** (R-HSA-397014)	198	**6**	**41 (21.0%)**	**11 (5.6%)**	**9.5E-04**	**63.4**	**32 (16.5%)**	**53.1**
**Gene Expression** (R-HSA-74160)	1755	**7**	**235 (13.7%)**	**76 (4.3%)**	**6.6E-03**	**55.7**	**233 (13.7%)**	**31.8**
**Metabolism** (R-HSA-1430728)	2035	**8**	**269 (13.5%)**	**100 (4.9%)**	**7.9E-03**	**69.1**	**311 (15.6%)**	**38.6**
**Extracellular matrix organization** (R-HSA-1474244)	295	**9**	**51 (17.6%)**	**23 (7.8%)**	**8.1E-03**	**52.9**	**43 (15.0%)**	**69.8**
**Immune System** (R-HSA-168256)	1950	**10**	**257 (13.5%)**	**95 (4.9%)**	**9.7E-03**	**68.1**	**197 (10.4%)**	**51.8**
**Cell Cycle** (R-HSA-1640170)	551	11	85 (15.7%)	34 (6.2%)	1.0E-02	54.1	71 (13.1%)	29.6
**DNA Repair** (R-HSA-73894)	323	12	54 (17.1%)	17 (5.3%)	1.1E-02	61.1	30 (9.5%)	30.0
**Organelle biogenesis and maintenance** (R-HSA-1852241)	310	13	50 (16.5%)	20 (6.5%)	2.7E-02	66	37 (12.2%)	35.1
**Developmental Biology** (R-HSA-1266738)	748	14	104 (14.1%)	33 (4.4%)	5.6E-02	57.7	77 (10.4%)	51.9
**Hemostasis** (R-HSA-109582)	693	15	97 (14.2%)	36 (5.2%)	5.6E-02	68	87 (13.0%)	51.7
**Cell-Cell communication** (R-HSA-1500931)	131	16	22 (17.1%)	7 (5.3%)	1.1E-01	68.2	19 (14.8%)	47.4
**Transport of small molecules** (R-HSA-382551)	628	17	75 (12.1%)	28 (4.5%)	4.4E-01	65.3	26 (4.2%)	53.8
**Neuronal System** (R-HSA-112316)	351	18	34 (9.8%)	13 (3.7%)	9.2E-01	47.1	20 (5.8%)	60.0
**Signal transduction** (R-HSA-162582)	2538	19	260 (10.4%)	93 (3.7%)	9.9E-01	58.1	160 (6.4%)	45.6

Bold text: highly significant (cluster q<0.01); text: significant (cluster q<0.05); text: cluster not significant, but contains significant (q<0.05) sub-pathways. The stable identifiers displayed next to the cluster names can be used to retrieve the full pathway information from the Reactome database

Table P2: Pathway analysis of Hepatic DEGs & DEPs detected after 0.1% DMSO exposure

Cluster name (stable identifier)	Set size	Ranking (DEGs)	Amount DEGs (%)	Amount DEGs (%)|log2FC|>1	q-value	% DEGs Down-regulated	Amount DEPs (%)	% DEPs Down-regulated
**Metabolism** (R-HSA-1430728)	**2035**	**1**	**472 (23.7%)**	**272 (13.4%)**	**9.1E-23**	**58.9**	**240 (12.1%)**	**52.5**
**Vesicle-mediated transport**(R-HSA-5653656)	**619**	**2**	**164 (26.8%)**	**96 (15.5%)**	**4.8E-11**	**51.5**	**52 (8.5%)**	**67.3**
**Extracellular matrix organization** (R-HSA-1474244)	**295**	**3**	**81 (28.0%)**	**55 (18.6%)**	**2.5E-06**	**77.8**	**No sub-pathways (q<0.05) detected**	
**Disease** (R-HSA-1643685)	**514**	**4**	**124 (24.5%)**	**77 (15.0%)**	**3.6E-06**	**61.3**	**39 (7.8%)**	**53.8**
**Immune System** (R-HSA-168256)	**1950**	**5**	**371 (19.5%)**	**220 (11.3%)**	**1.0E-05**	**58.5**	**124 (6.5%)**	**56.5**
**Muscle contraction** (R-HSA-397014)	**198**	**6**	**58 (29.7%)**	**40 (20.2%)**	**1.4E-05**	**82.8**	**15 (7.7%)**	**46.7**
**Metabolism of proteins** (R-HSA-392499)	**1506**	**7**	**291 (19.7%)**	**176 (11.7%)**	**9.3E-05**	**59.9**	**145 (9.9%)**	**60.0**
**Cellular responses to stress** (R-HSA-2262752)	**393**	**8**	**88 (22.7%)**	**59 (15.0%)**	**2.1E-03**	**69.3**	**36 (9.3%)**	**69.4**
**Hemostasis**(R-HSA-109582)	**693**	**9**	**141 (20.6%)**	**88 (12.7%)**	**3.2E-03**	**56.7**	**43 (6.4%)**	**60.5**
**Developmental Biology** (R-HSA-1266738)	**748**	**10**	**149 (20.2%)**	**85 (11.4%)**	**4.9E-03**	**69.1**	**49 (6.6%)**	**46.9**
**Cell-Cell communication** (R-HSA-1500931)	**131**	**11**	**35 (27.1%)**	**21 (16.0%)**	**6.8E-03**	**62.9**	**No sub-pathways (q<0.05) detected**	
**Programmed Cell Death** (R-HSA-5357801)	125	12	33 (26.8%)	15 (12.0%)	1.0E-02	54.5	18 (14.8%)	72.2
**Transport of small molecules** (R-HSA-382551)	628	13	114 (18.4%)	75 (11.9%)	1.2E-01	58.8	21 (3.4%)	42.9
**Signal transduction** (R-HSA-162582)	2538	14	377 (15.1%)	257 (10.1%)	8.5E-01	64.5	105 (4.2%)	67.6
**Neuronal system** (R-HSA-112316)	351	15	44 (12.6%)	31 (8.8%)	9.8E-01	68.2	12 (3.5%)	50.0
**Gene expression** (R-HSA-74160)	1755	16	239 (14.0%)	145 (8.3%)	1.0E+0	55.6	115 (6.8%)	60.0

Bold text: highly significant (cluster q<0.01); text: significant (cluster q<0.05); text: cluster not significant, but contains significant (q<0.05) sub-pathways. The stable identifiers displayed next to the cluster names can be used to retrieve the full pathway information from the Reactome database

Interestingly, Table P3 indicates that while the majority of DEGs was downregulated in both tissue types, DEPs displayed tissue-specific changes with almost all cardiac DEPs being increased and all hepatic DEPs being decreased. Unfortunately, the proteome coverage was too limited for in depth analysis.

Table P3: Pathways related to transcriptional regulation

		Cardiac					Hepatic				
	Set size	DEGs	q-value	% DEGs down-regulated	DEPs	% DEPs down-regulated	DEGs	q-value	% DEGs down-regulated	DEPs	% DEPs down-regulated
**Gene silencing by RNAs**	134	35 (26.7%)	3.8E-05	74.3	32 (24.4%)	0%	25 (19.1%)	3.6E-01	72.0	17 (13.0%)	100%
**Transcriptional regulation by small RNAs**	108	32 (30.2%)	7.6E-06	75.0	31 (29.0%)	0%	24 (22.6%)	1.4E-01	75.0	17 (15.9%)	100%
**MicroRNA biogenesis**	13	5 (41.7%)	4.0E-02	60.0	2 (18.2%)	0%	1 (8.3%)	9.6E-01	0.0	1 (9.1%)	100%
**Epigenetic regulation of gene expression**	154	31 (20.5%)	6.8E-03	61.3	34 (22.7%)	5.9%	27 (17.9%)	4.5E-01	77.7	17 (11.3%)	100%
**DNA methylation**	68	18 (27.3%)	3.4E-03	66.6	30 (44.8%)	0%	13 (19.7%)	4.4E-01	92.3	16 (23.9%)	100%

Supplementary figures

Supplementary figure 1: PCA plot to detect outliers. Samples were exposed to medium with (red) or without (black) 0.1% DMSO. In cardiac samples. Four outliers (names indicated) were detected in the cardiac dataset, which were deleted from the analysis. Two most distant hepatic samples (names indicated) were not considered as outliers.

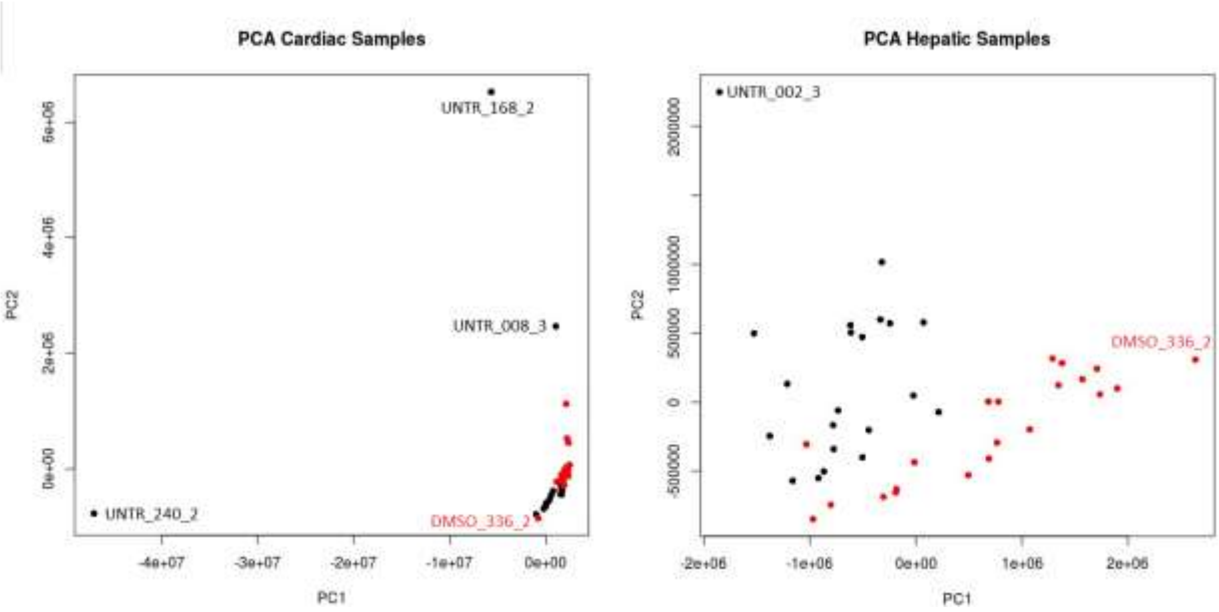

Supplementary figure 2: Changes in microtissue ATP content over time. Values at T0 were set to 100%. Cardiac samples are indicated with squares and hepatic samples with circles. ATP percentage per microtissue is depicted in red for DMSO exposed samples and blue for UNTR samples. Green shows the effect of DMSO (corrected for the control by subtracting values of the UNTR MTs).

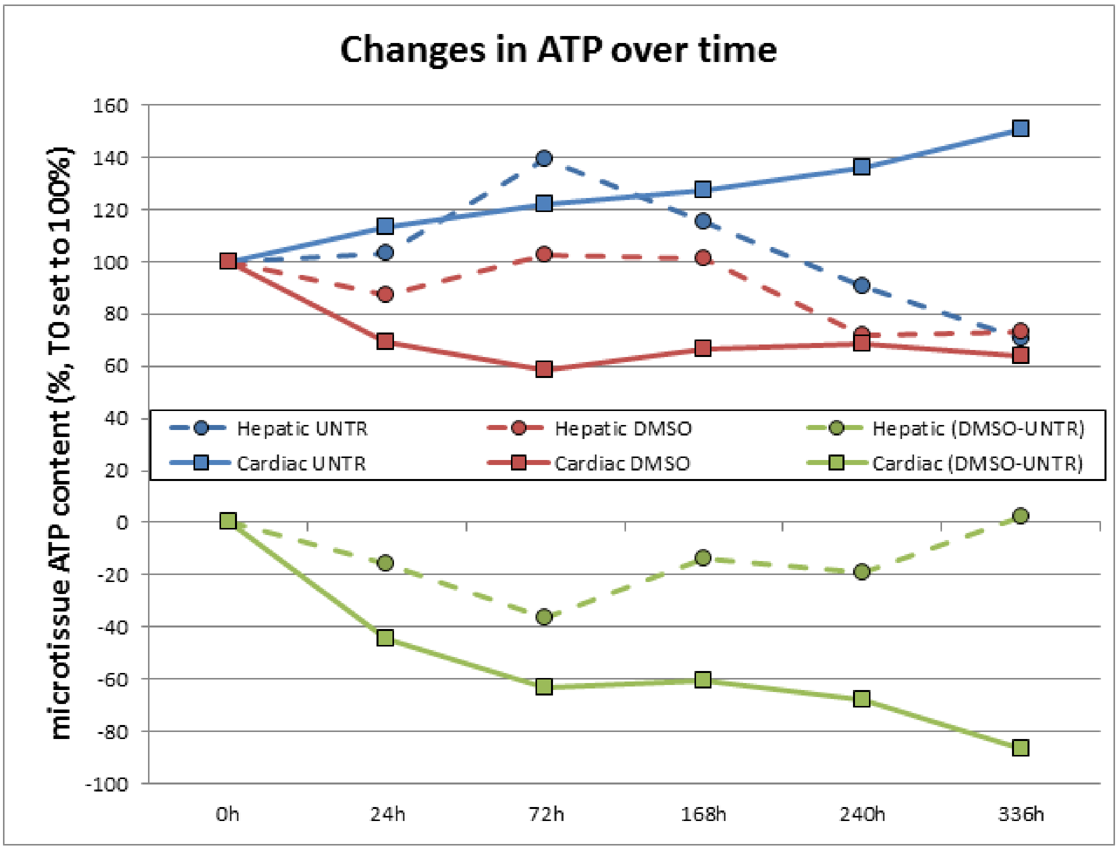

Supplementary figure 3: Indication of biological effects due to miRNA changes.
Pathway overrepresentation using gene targets of DE miRNA. Dark colored pathways are significantly overrepresented in the dataset

A) Hepatic

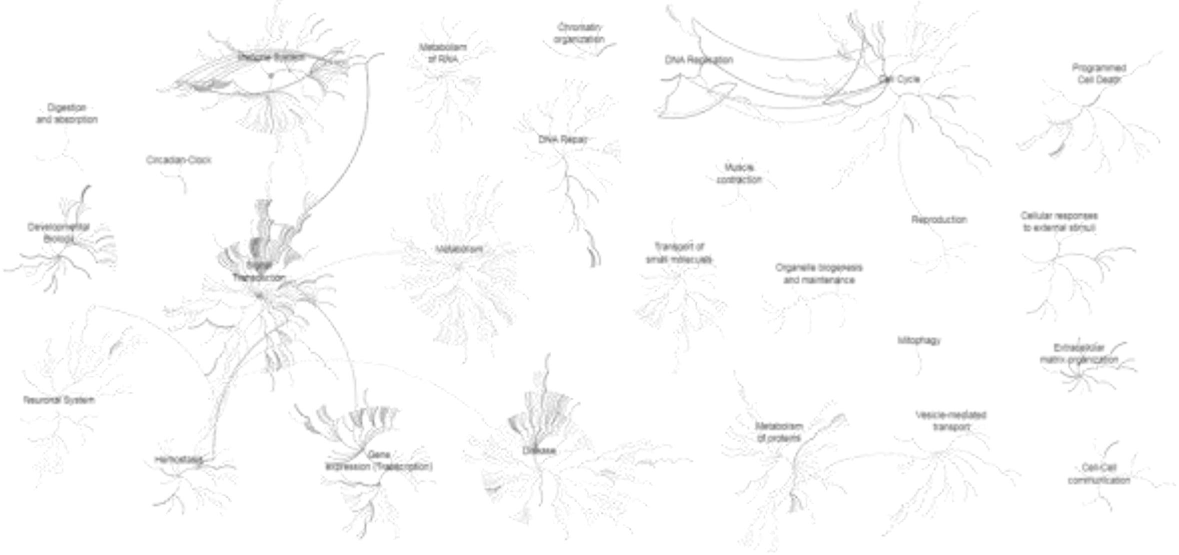

B) Cardiac

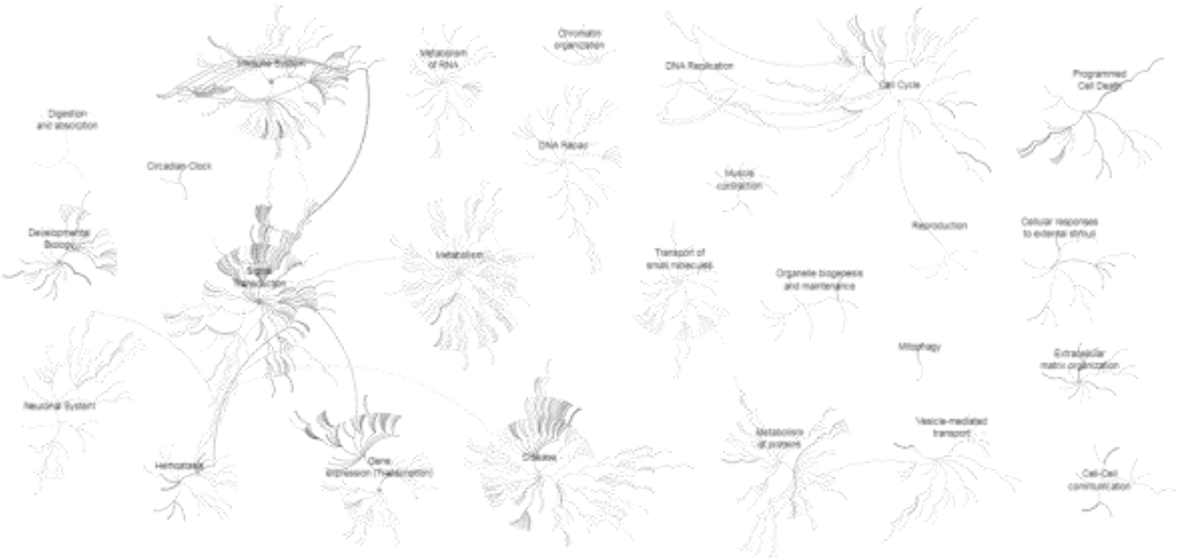

Supplementary tables

Supplementary table 1: Hierarchically ordered overrepresented pathways (using DEGs) in cardiac MTs after 0.1% DMSO exposure

Cluster	Pathway	Set size	Amount DEGs (%)	q-value	Amount DEGs with |log2FC|>1 (%)	q-value
Cellular responses to stress	Cellular responses to stress	393	91 (23.5%)	2.25E-09	26 (7.6%)	0.168
Cellular responses to stress	Oxygen-dependent proline hydroxylation of Hypoxia-inducible Factor Alpha	21	11 (52.4%)	1.22E-04	3 (14.3%)	0.296
Cellular responses to stress	Cellular Senescence	192	42 (22.5%)	2.55E-04	16 (8.6%)	0.19
Cellular responses to stress	Regulation of Hypoxia-inducible Factor (HIF) by oxygen	23	11 (47.8%)	3.28E-04	3 (13.0%)	0.307
Cellular responses to stress	Cellular response to hypoxia	23	11 (47.8%)	3.28E-04	3 (13.0%)	0.307
Cellular responses to stress	Senescence-Associated Secretory Phenotype (SASP)	113	28 (25.5%)	5.10E-04	11 (10.0%)	0.19
Cellular responses to stress	Oxidative Stress Induced Senescence	129	27 (21.6%)	6.57E-03	12 (9.6%)	0.19
Cellular responses to stress	Cellular response to heat stress	100	22 (22.0%)	1.26E-02	7 (7.1%)	0.363
Cellular responses to stress	Macroautophagy	56	14 (25.0%)	2.27E-02	2 (3.5%)	0.815
Cellular responses to stress	HSF1 activation	31	9 (29.0%)	3.50E-02	3 (9.7%)	0.375
Cellular responses to stress	Regulation of HSF1-mediated heat shock response	81	17 (21.0%)	4.41E-02	7 (8.6%)	0.3
Disease	Disease	514	109 (21.5%)	4.48E-09	34 (6.7%)	0.19
Disease	Infectious disease	254	64 (25.6%)	2.73E-08	22 (8.8%)	0.109
Disease	HIV Infection	196	49 (25.5%)	2.37E-06	17 (8.7%)	0.183
Disease	Elongation arrest and recovery	55	21 (40.4%)	4.95E-06	NA	NA
Disease	Influenza Infection	63	23 (36.5%)	9.41E-06	6 (9.5%)	0.294
Disease	Influenza Life Cycle	53	20 (37.7%)	2.79E-05	5 (9.4%)	0.307
Disease	Tat-mediated HIV elongation arrest and recovery	40	16 (43.2%)	3.75E-05	5 (13.9%)	0.229
Disease	Pausing and recovery of Tat-mediated HIV elongation	40	16 (43.2%)	3.75E-05	5 (13.9%)	0.229
Disease	HIV Life Cycle	155	38 (25.0%)	6.57E-05	16 (10.4%)	0.095
Disease	HIV elongation arrest and recovery	42	16 (41.0%)	6.93E-05	5 (13.2%)	0.237
Disease	Pausing and recovery of HIV elongation	42	16 (41.0%)	6.93E-05	5 (13.2%)	0.237
Disease	Late Phase of HIV Life Cycle	142	35 (25.2%)	1.14E-04	15 (10.6%)	0.0979
Disease	Abortive elongation of HIV-1 transcript in the absence of Tat	25	12 (48.0%)	1.42E-04	3 (13.0%)	0.307
Disease	Transcription of the HIV genome	79	22 (28.9%)	4.74E-04	9 (12.0%)	0.183
Disease	Host Interactions of HIV factors	91	24 (27.3%)	5.30E-04	7 (7.9%)	0.315
Disease	Viral Messenger RNA Synthesis	42	15 (35.7%)	5.30E-04	3 (7.1%)	0.501
Disease	HIV Transcription Initiation	47	16 (34.0%)	5.38E-04	6 (12.8%)	0.229
Disease	RNA Polymerase II HIV Promoter Escape	47	16 (34.0%)	5.38E-04	6 (12.8%)	0.229
Disease	Influenza Viral RNA Transcription and Replication	44	15 (34.1%)	8.97E-04	3 (6.8%)	0.518
Disease	Tat-mediated elongation of the HIV-1 transcript	52	16 (32.7%)	8.98E-04	5 (10.4%)	0.294
Disease	HIV Transcription Elongation	52	16 (32.7%)	8.98E-04	5 (10.4%)	0.294
Disease	Formation of HIV-1 elongation complex containing HIV-1 Tat	52	16 (32.7%)	8.98E-04	5 (10.4%)	0.294
Disease	RNA Pol II CTD phosphorylation and interaction with CE during HIV infection	27	11 (40.7%)	1.24E-03	2 (7.4%)	0.547
Disease	Formation of HIV elongation complex in the absence of HIV Tat	54	16 (31.4%)	1.41E-03	5 (10.0%)	0.3
Disease	Formation of the HIV-1 Early Elongation Complex	35	12 (34.3%)	3.39E-03	3 (9.1%)	0.398
Disease	Signaling by BRAF and RAF fusions	61	15 (25.4%)	1.47E-02	3 (5.2%)	0.65

(table continued) Cluster	Pathway	Set size	Amount DEGs (%)	q-value	Amount DEGs with |log2FC|>1 (%)	q-value
Disease	Vif-mediated degradation of APOBEC3G	10	5 (50.0%)	2.02E-02	3 (30.0%)	0.19
Disease	Oncogenic MAPK signaling	64	15 (24.2%)	2.30E-02	3 (4.9%)	0.675
Disease	Diseases of signal transduction	251	42 (17.0%)	2.96E-02	13 (5.3%)	0.51
Disease	InlA-mediated entry of Listeria monocytogenes into host cells	8	4 (50.0%)	4.15E-02	2 (25.0%)	0.268
Vesicle-mediated transport	Vesicle-mediated transport	619	122 (20.0%)	2.70E-08	48 (7.7%)	0.0438
Vesicle-mediated transport	Membrane Trafficking	580	117 (20.4%)	1.98E-08	45 (7.7%)	0.0495
Vesicle-mediated transport	Golgi-to-ER retrograde transport	111	35 (31.8%)	7.58E-07	13 (11.2%)	0.11
Metabolism of proteins	ER to Golgi Anterograde Transport	134	38 (29.0%)	2.35E-06	10 (7.3%)	0.307
Metabolism of proteins	COPI-mediated anterograde transport	79	27 (35.1%)	2.72E-06	6 (7.2%)	0.376
Vesicle-mediated transport	Intra-Golgi and retrograde Golgi-to-ER traffic	181	46 (25.6%)	4.95E-06	16 (8.6%)	0.19
Vesicle-mediated transport	COPI-independent Golgi-to-ER retrograde traffic	28	12 (42.9%)	4.74E-04	4 (11.8%)	0.296
Vesicle-mediated transport	COPI-dependent Golgi-to-ER retrograde traffic	83	23 (28.0%)	5.08E-04	9 (11.0%)	0.19
Vesicle-mediated transport	Clathrin derived vesicle budding	73	19 (26.4%)	3.53E-03	9 (12.5%)	0.167
Vesicle-mediated transport	trans-Golgi Network Vesicle Budding	73	19 (26.4%)	3.53E-03	9 (12.5%)	0.167
Vesicle-mediated transport	Golgi Associated Vesicle Biogenesis	57	16 (28.6%)	3.82E-03	8 (14.3%)	0.16
Metabolism of proteins	COPII (Coat Protein 2) Mediated Vesicle Transport	70	18 (26.1%)	5.32E-03	NA	NA
Metabolism of proteins	Metabolism of proteins	1506	236 (16.0%)	9.30E-07	114 (5.7%)	0.095
Metabolism of proteins	Asparagine N-linked glycosylation	283	64 (22.9%)	2.07E-06	19 (6.7%)	0.268
Metabolism of proteins	ER to Golgi Anterograde Transport	134	38 (29.0%)	2.35E-06	10 (7.3%)	0.307
Metabolism of proteins	COPI-mediated anterograde transport	79	27 (35.1%)	2.72E-06	6 (7.2%)	0.376
Metabolism of proteins	Transport to the Golgi and subsequent modification	165	42 (25.9%)	1.00E-05	10 (6.0%)	0.421
Metabolism of proteins	UCH proteinases	107	27 (26.5%)	3.92E-04	10 (9.8%)	0.229
Metabolism of proteins	Post-translational protein modification	1026	156 (15.4%)	5.22E-04	78 (5.7%)	0.19
Metabolism of proteins	COPII (Coat Protein 2) Mediated Vesicle Transport	70	18 (26.1%)	5.32E-03	NA	NA
Metabolism of proteins	Amyloid fiber formation	83	20 (24.7%)	5.62E-03	9 (11.1%)	0.19
Metabolism of proteins	Protein folding	106	22 (20.8%)	2.42E-02	4 (3.9%)	0.787
Metabolism of proteins	Mitochondrial protein import	63	15 (23.8%)	2.64E-02	4 (6.2%)	0.536
Metabolism of proteins	E3 ubiquitin ligases ubiquitinate target proteins	16	6 (37.5%)	3.50E-02	7 (11.9%)	0.229
Metabolism of proteins	Chaperonin-mediated protein folding	100	20 (20.0%)	4.27E-02	4 (4.1%)	0.758
Metabolism of proteins	Protein ubiquitination	39	10 (26.3%)	4.27E-02	8 (10.1%)	0.229
Metabolism of proteins	Cooperation of PDCL (PhLP1) and TRiC/CCT in G-protein beta folding	44	11 (25.0%)	4.41E-02	3 (7.0%)	0.516
Metabolism of proteins	N-glycan trimming in the ER and Calnexin/Calreticulin cycle	34	9 (27.3%)	4.68E-02	3 (9.1%)	0.398
Chromatin organization	Chromatin organization	274	58 (21.3%)	5.23E-05	19 (7.0%)	0.248
Chromatin organization	Chromatin modifying enzymes	274	58 (21.3%)	5.23E-05	19 (7.0%)	0.248
Chromatin organization	HDACs deacetylate histones	94	22 (23.4%)	6.53E-03	8 (8.5%)	0.294
Chromatin organization	RMTs methylate histone arginines	74	17 (23.0%)	2.30E-02	6 (8.1%)	0.33
Chromatin organization	HATs acetylate histones	143	27 (18.9%)	3.20E-02	7 (4.9%)	0.62
Muscle contraction	Muscle contraction	198	41 (21.0%)	9.53E-04	11 (5.6%)	0.453
Muscle contraction	Striated Muscle Contraction	35	16 (45.7%)	1.80E-05	4 (11.4%)	0.3
Muscle contraction	Smooth Muscle Contraction	36	14 (40.0%)	3.09E-04	4 (11.4%)	0.3
Gene expression	Gene Expression	1755	235 (13.7%)	6.57E-03	NA	NA
Gene expression	Transcriptional Regulation by TP53	376	84 (22.7%)	2.97E-08	28 (7.6%)	0.167
Gene expression	Transcriptional regulation by small RNAs	108	32 (30.2%)	7.63E-06	8 (7.5%)	0.315

(table continued)<br>Cluster	Pathway	Set size	Amount DEGs (%)	q-value	Amount DEGs with |log2FC|>1 (%)	q-value
Gene expression	RNA Polymerase II Pre-transcription Events	90	27 (31.0%)	3.09E-05	9 (10.5%)	0.218
Gene expression	Gene Silencing by RNA	134	35 (26.7%)	3.75E-05	10 (7.5%)	0.3
Gene expression	TP53 Regulates Metabolic Genes	86	26 (30.6%)	5.23E-05	6 (7.0%)	0.398
Gene expression	mRNA Splicing - Minor Pathway	53	19 (35.8%)	7.98E-05	4 (7.7%)	0.421
Gene expression	Formation of RNA Pol II elongation complex	67	21 (32.8%)	1.15E-04	5 (7.9%)	0.362
Gene expression	RNA Polymerase II Transcription Elongation	67	21 (32.8%)	1.15E-04	5 (7.9%)	0.362
Gene expression	RNA Polymerase II Transcription	163	38 (24.2%)	1.15E-04	58 (4.7%)	0.498
Gene expression	Processing of Capped Intron-Containing Pre-mRNA	248	50 (20.7%)	3.92E-04	17 (7.1%)	0.268
Gene expression	mRNA Splicing - Major Pathway	185	40 (22.2%)	4.55E-04	15 (8.4%)	0.213
Gene expression	RNA Polymerase II Promoter Escape	46	16 (34.8%)	4.92E-04	6 (13.0%)	0.229
Gene expression	mRNA Splicing	193	41 (21.8%)	5.08E-04	15 (8.1%)	0.229
Gene expression	RNA polymerase II transcribes snRNA genes	80	22 (28.6%)	5.22E-04	9 (11.7%)	0.19
Gene expression	RNA Polymerase II Transcription Pre-Initiation And Promoter Opening	47	16 (34.0%)	5.38E-04	6 (12.8%)	0.229
Gene expression	RNA Polymerase II Transcription Initiation	47	16 (34.0%)	5.38E-04	6 (12.8%)	0.229
Gene expression	RNA Polymerase II Transcription Initiation And Promoter Clearance	47	16 (34.0%)	5.38E-04	6 (12.8%)	0.229
Gene expression	RNA Pol II CTD phosphorylation and interaction with CE	27	11 (40.7%)	1.24E-03	2 (7.4%)	0.547
Gene expression	mRNA Capping	29	11 (37.9%)	2.36E-03	2 (6.9%)	0.572
Gene expression	ERCC6 (CSB) and EHMT2 (G9a) positively regulate rRNA expression	79	20 (26.0%)	3.29E-03	8 (10.3%)	0.229
Gene expression	DNA methylation	68	18 (27.3%)	3.37E-03	7 (10.6%)	0.237
Gene expression	Formation of the Early Elongation Complex	35	12 (34.3%)	3.39E-03	3 (9.1%)	0.398
Gene expression	TP53 Regulates Transcription of DNA Repair Genes	72	18 (26.5%)	4.55E-03	5 (7.5%)	0.392
Gene expression	Deadenylation of mRNA	25	9 (39.1%)	5.52E-03	3 (12.0%)	0.315
Gene expression	RNA Polymerase I Promoter Opening	66	17 (26.6%)	5.74E-03	6 (9.4%)	0.296
Gene expression	Epigenetic regulation of gene expression	154	31 (20.5%)	6.81E-03	10 (6.7%)	0.346
Gene expression	RNA Polymerase I Transcription	115	25 (22.1%)	6.87E-03	9 (8.0%)	0.296
Gene expression	Positive epigenetic regulation of rRNA expression	111	24 (22.0%)	9.02E-03	8 (7.4%)	0.327
Gene expression	PRC2 methylates histones and DNA	77	18 (24.3%)	1.06E-02	7 (9.5%)	0.268
Gene expression	RNA Polymerase I Promoter Clearance	113	24 (21.6%)	1.09E-02	8 (7.2%)	0.339
Gene expression	Negative epigenetic regulation of rRNA expression	113	24 (21.6%)	1.09E-02	7 (6.4%)	0.43
Gene expression	RNA Polymerase I, RNA Polymerase III, and Mitochondrial Transcription	153	30 (20.0%)	1.12E-02	NA	NA
Gene expression	SIRT1 negatively regulates rRNA Expression	72	17 (24.3%)	1.33E-02	7 (10.1%)	0.257
Gene expression	NoRC negatively regulates rRNA expression	110	23 (21.3%)	1.53E-02	6 (5.6%)	0.547
Gene expression	B-WICH complex positively regulates rRNA expression	96	20 (21.3%)	2.64E-02	6 (6.5%)	0.448
Gene expression	Small interfering RNA (siRNA) biogenesis	7	4 (57.1%)	2.67E-02	2 (22.2%)	0.294
Gene expression	Deadenylation-dependent mRNA decay	57	13 (24.1%)	3.55E-02	3 (5.4%)	0.635
Gene expression	RNA Polymerase I Chain Elongation	94	19 (20.7%)	3.79E-02	6 (6.6%)	0.432
Gene expression	MicroRNA (miRNA) biogenesis	13	5 (41.7%)	3.98E-02	2 (16.7%)	0.315
Metabolism	Metabolism	2035	269 (13.5%)	7.85E-03	95 (4.9%)	0.352
Metabolism	The citric acid (TCA) cycle and respiratory electron transport	171	58 (34.7%)	1.26E-12	15 (8.7%)	0.19
Metabolism	Respiratory electron transport, ATP synthesis by chemiosmotic coupling, and heat production by uncoupling proteins.	126	47 (38.2%)	5.19E-12	12 (9.8%)	0.19
Metabolism	Respiratory electron transport	103	37 (37.0%)	4.48E-09	9 (9.0%)	0.257
Metabolism	Formation of ATP by chemiosmotic coupling	18	10 (55.6%)	1.60E-04	3 (16.7%)	0.268

(table continued) Cluster	Pathway	Set size	Amount DEGs (%)	q-value	Amount DEGs with |log2FC|>1 (%)	q-value
Metabolism	Glucagon-like Peptide-1 (GLP1) regulates insulin secretion	35	13 (37.1%)	9.52E-04	5 (14.3%)	0.229
Metabolism	Regulation of insulin secretion	70	19 (27.1%)	2.61E-03	6 (8.6%)	0.307
Metabolism	Glucose metabolism	77	20 (26.3%)	2.81E-03	7 (7.8%)	0.324
Metabolism	Glycolysis	33	11 (34.4%)	5.27E-03	4 (5.7%)	0.573
Metabolism	Adrenaline,noradrenaline inhibits insulin secretion	23	9 (39.1%)	5.52E-03	3 (13.0%)	0.307
Metabolism	Pyruvate metabolism and Citric Acid (TCA) cycle	49	14 (29.2%)	6.15E-03	5 (9.3%)	0.307
Metabolism	Integration of energy metabolism	94	22 (23.4%)	6.53E-03	6 (6.4%)	0.453
Metabolism	Keratan sulfate degradation	5	4 (80.0%)	6.78E-03	2 (40.0%)	0.229
Metabolism	Metabolism of carbohydrates	288	50 (17.5%)	9.83E-03	13 (4.9%)	0.572
Metabolism	Glucagon signaling in metabolic regulation	33	10 (30.3%)	1.98E-02	4 (12.1%)	0.294
Metabolism	Hyaluronan uptake and degradation	10	5 (50.0%)	2.02E-02	2 (20.0%)	0.3
Metabolism	Citric acid cycle (TCA cycle)	19	7 (36.8%)	2.37E-02	2 (9.1%)	0.473
Metabolism	CS/DS degradation	7	4 (57.1%)	2.67E-02	2 (28.6%)	0.268
Metabolism	Regulation of lipid metabolism by Peroxisome proliferator-activated receptor alpha (PPARalpha)	20	7 (35.0%)	2.94E-02	1 (5.0%)	0.732
Metabolism	Hyaluronan metabolism	12	5 (41.7%)	3.98E-02	2 (16.7%)	0.315
Metabolism	Complex I biogenesis	57	13 (23.6%)	4.00E-02	2 (3.6%)	0.802
Metabolism	Gluconeogenesis	33	9 (28.1%)	4.01E-02	5 (14.7%)	0.229
Metabolism	Pyruvate metabolism	28	8 (29.6%)	4.27E-02	3 (10.0%)	0.363
Extracellular matrix organization	Extracellular matrix organization	295	51 (17.6%)	8.08E-03	24 (8.2%)	0.143
Extracellular matrix organization	Collagen formation	94	22 (23.9%)	5.25E-03	12 (13.2%)	0.061
Extracellular matrix organization	Assembly of collagen fibrils and other multimeric structures	49	12 (25.0%)	3.51E-02	7 (14.6%)	0.168
Extracellular matrix organization	Degradation of the extracellular matrix	107	21 (20.0%)	3.78E-02	9 (8.6%)	0.268
Extracellular matrix organization	Collagen biosynthesis and modifying enzymes	70	15 (21.7%)	4.74E-02	8 (11.9%)	0.19
Immune System	Immune System	1950	257 (13.5%)	9.66E-03	94 (5.2%)	0.268
Immune System	Neutrophil degranulation	497	104 (21.5%)	1.12E-08	42 (8.7%)	0.0184
Immune System	Antigen Presentation: Folding, assembly and peptide loading of class I MHC	24	11 (45.8%)	4.74E-04	3 (12.5%)	0.307
Immune System	Innate Immune System	1309	188 (14.7%)	1.01E-03	61 (5.8%)	0.229
Immune System	MAPK1 (ERK2) activation	9	5 (55.6%)	1.22E-02	2 (22.2%)	0.294
Immune System	RAF-independent MAPK1/3 activation	23	8 (34.8%)	1.98E-02	3 (13.0%)	0.307
Immune System	MAPK3 (ERK1) activation	10	5 (50.0%)	2.02E-02	2 (20.0%)	0.3
Immune System	Cytosolic sensors of pathogen-associated DNA	50	13 (26.0%)	2.19E-02	5 (9.8%)	0.306
Immune System	TRIF-mediated TLR3/TLR4 signaling	101	21 (20.8%)	2.67E-02	NA	NA
Immune System	MyD88-independent TLR3/TLR4 cascade	101	21 (20.8%)	2.67E-02	NA	NA
Immune System	Toll Like Receptor 3 (TLR3) Cascade	101	21 (20.8%)	2.67E-02	7 (7.2%)	0.352
Immune System	Interleukin-6 signaling	11	5 (45.5%)	2.90E-02	2 (18.2%)	0.307
Immune System	STING mediated induction of host immune responses	16	6 (37.5%)	3.50E-02	1 (6.2%)	0.675
Cell Cycle	Cell Cycle	551	85 (15.7%)	1.03E-02	39 (7.0%)	0.158
Cell Cycle	Cell Cycle, Mitotic	468	76 (16.5%)	5.27E-03	38 (8.0%)	0.0552
Cell Cycle	M Phase	267	48 (18.3%)	5.46E-03	29 (8.6%)	0.061
Cell Cycle	Mitotic Prophase	143	29 (20.6%)	9.17E-03	12 (8.5%)	0.229
Cell Cycle	Meiotic recombination	65	16 (25.4%)	1.14E-02	6 (9.5%)	0.294
Cell Cycle	Meiosis	79	18 (23.7%)	1.33E-02	7 (9.2%)	0.276

(table continued)<br>Cluster	Pathway	Set size	Amount DEGs (%)	q-value	Amount DEGs with |log2FC|>1 (%)	q-value
Cell Cycle	Regulation of PLK1 Activity at G2/M Transition	90	19 (21.6%)	2.67E-02	8 (9.2%)	0.268
Cell Cycle	APC-Cdc20 mediated degradation of Nek2A	26	8 (32.0%)	2.96E-02	4 (16.0%)	0.229
Cell Cycle	Mitotic G2-G2/M phases	138	26 (19.1%)	3.15E-02	12 (8.6%)	0.229
Cell Cycle	Autodegradation of Cdh1 by Cdh1:APC/C	22	7 (33.3%)	3.67E-02	3 (14.3%)	0.296
Cell Cycle	Cdc20:Phospho-APC/C mediated degradation of Cyclin A	28	8 (29.6%)	4.27E-02	4 (14.8%)	0.248
Cell Cycle	G2/M Transition	136	25 (18.7%)	4.35E-02	11 (8.0%)	0.268
Cell Cycle	APC/C:Cdc20 mediated degradation of Securin	23	7 (31.8%)	4.42E-02	3 (13.6%)	0.3
Cell Cycle	Condensation of Prophase Chromosomes	77	16 (21.3%)	4.60E-02	6 (8.0%)	0.335
DNA Repair	DNA Repair	323	54 (17.1%)	1.07E-02	17 (5.4%)	0.453
DNA repair	Transcription-Coupled Nucleotide Excision Repair (TC-NER)	80	22 (27.5%)	8.80E-04	6 (7.4%)	0.364
DNA repair	Nucleotide Excision Repair	113	27 (24.1%)	1.41E-03	8 (7.1%)	0.346
DNA repair	Dual incision in TC-NER	68	19 (27.9%)	1.80E-03	5 (7.2%)	0.409
DNA repair	Translesion synthesis by POLK	17	8 (47.1%)	2.99E-03	5 (29.4%)	0.061
DNA repair	Formation of TC-NER Pre-Incision Complex	56	16 (28.6%)	3.82E-03	4 (7.0%)	0.457
DNA repair	Gap-filling DNA repair synthesis and ligation in TC-NER	67	18 (26.9%)	3.85E-03	5 (7.4%)	0.4
DNA repair	Translesion synthesis by REV1	16	7 (43.8%)	9.23E-03	5 (31.2%)	0.061
DNA repair	Translesion synthesis by POLI	17	7 (41.2%)	1.26E-02	5 (29.4%)	0.061
DNA repair	Recruitment and ATM-mediated phosphorylation of repair and signaling proteins at DNA double strand breaks	77	17 (22.4%)	2.74E-02	8 (10.5%)	0.229
DNA repair	DNA Double Strand Break Response	78	17 (22.1%)	3.05E-02	8 (10.4%)	0.229
DNA repair	DNA Double-Strand Break Repair	171	30 (18.0%)	3.98E-02	10 (6.0%)	0.421
DNA repair	DNA Damage Recognition in GG-NER	39	10 (26.3%)	4.27E-02	5 (13.2%)	0.237
Organelle biogenesis and maintenance	Organelle biogenesis and maintenance	310	50 (16.5%)	2.67E-02	16 (6.8%)	0.294
	no sub-pathways detected				NA	NA
Developmental Biology	Developmental Biology	748	104 (14.1%)	5.57E-02	26 (4.2%)	0.748
Developmental Biology	Semaphorin interactions	68	22 (32.8%)	7.49E-05	4 (6.2%)	0.525
Developmental Biology	Sema4D in semaphorin signaling	27	12 (44.4%)	3.30E-04	3 (12.5%)	0.307
Developmental Biology	Sema4D induced cell migration and growth-cone collapse	24	10 (41.7%)	1.88E-03	1 (5.0%)	0.732
Developmental Biology	Axon guidance	487	81 (16.8%)	2.13E-03	17 (4.8%)	0.584
Developmental Biology	SEMA3A-Plexin repulsion signaling by inhibiting Integrin adhesion	15	7 (50.0%)	4.16E-03	1 (7.1%)	0.649
Developmental Biology	CRMPs in Sema3A signaling	16	6 (37.5%)	3.50E-02	1 (6.2%)	0.675
Developmental Biology	Sema3A PAK dependent Axon repulsion	16	6 (37.5%)	3.50E-02	1 (6.2%)	0.675
Developmental Biology	Transcriptional regulation of white adipocyte differentiation	50	12 (25.0%)	3.51E-02	7 (14.9%)	0.167
Hemostasis	Hemostasis	693	97 (14.2%)	5.63E-02	36 (5.4%)	0.332
Hemostasis	Response to elevated platelet cytosolic Ca2+	138	31 (22.8%)	1.41E-03	12 (9.0%)	0.229
Hemostasis	Platelet degranulation	133	30 (22.9%)	1.61E-03	11 (8.5%)	0.237
Hemostasis	Platelet activation, signaling and aggregation	283	49 (17.4%)	1.16E-02	18 (6.9%)	0.268
Hemostasis	Prostacyclin signalling through prostacyclin receptor	19	7 (36.8%)	2.37E-02	3 (15.8%)	0.276
Hemostasis	Platelet homeostasis	92	19 (20.9%)	3.50E-02	5 (5.6%)	0.566
Cell-Cell communication	Cell-Cell communication	131	22 (17.1%)	1.08E-01	7 (5.7%)	0.516
Cell-Cell communication	Nectin/Necl trans heterodimerization	7	4 (57.1%)	2.67E-02	1 (14.3%)	0.488
Transport of small molecules	(Transmembrane) transport of small molecules	628	75 (12.1%)	4.44E-01	NA	NA
Transport of small molecules	Vasopressin regulates renal water homeostasis	40	11 (27.5%)	2.64E-02	4 (10.0%)	0.316

(table continued)<br>Cluster	Pathway	Set size	Amount DEGs (%)	q-value	Amount DEGs with |log2FC|>1 (%)	q-value
Neuronal System	Neuronal System	351	34 (9.8%)	9.20E-01	15 (4.1%)	0.775
Neuronal System	Presynaptic function of Kainate receptors	21	8 (38.1%)	1.12E-02	4 (19.0%)	0.213
Neuronal System	Activation of Kainate Receptors upon glutamate binding	32	9 (28.1%)	4.01E-02	4 (13.3%)	0.268
Signal transduction	Signal transduction	2538	260 (10.4%)	9.94E-01	103 (3.9%)	0.939
Signal transduction	RHO GTPases activate PKNs	96	28 (29.8%)	4.20E-05	7 (7.4%)	0.346
Signal transduction	FGFR2 alternative splicing	29	13 (48.1%)	6.93E-05	3 (11.1%)	0.339
Signal transduction	RHO GTPase Effectors	299	61 (20.7%)	7.04E-05	18 (6.0%)	0.332
Signal transduction	Signaling by Wnt	286	56 (20.1%)	3.28E-04	18 (6.5%)	0.296
Signal transduction	TCF dependent signaling in response to WNT	195	41 (21.8%)	5.08E-04	12 (6.4%)	0.346
Signal transduction	Formation of the beta-catenin:TCF transactivating complex	95	25 (26.9%)	5.08E-04	9 (9.7%)	0.229
Signal transduction	RHO GTPases activate PAKs	23	10 (43.5%)	1.33E-03	2 (9.5%)	0.453
Signal transduction	Signaling by Rho GTPases	434	74 (17.4%)	1.58E-03	25 (5.8%)	0.313
Signal transduction	RHO GTPases activate CIT	17	8 (47.1%)	2.99E-03	1 (5.3%)	0.719
Signal transduction	Signaling by FGFR2	75	19 (26.0%)	4.11E-03	5 (6.8%)	0.442
Signal transduction	G beta:gamma signalling through PLC beta	20	8 (40.0%)	8.48E-03	4 (20.0%)	0.199
Signal transduction	Activated PKN1 stimulates transcription of AR (androgen receptor) regulated genes KLK2 and KLK3	70	17 (25.0%)	1.04E-02	6 (8.8%)	0.307
Signal transduction	Signaling by FGFR	89	20 (23.0%)	1.18E-02	6 (6.9%)	0.407
Signal transduction	RHO GTPases Activate ROCKs	17	7 (41.2%)	1.26E-02	1 (5.3%)	0.719
Signal transduction	NOTCH1 Intracellular Domain Regulates Transcription	48	13 (27.1%)	1.54E-02	4 (8.5%)	0.374
Signal transduction	Opioid Signalling	85	19 (22.6%)	1.74E-02	7 (8.6%)	0.3
Signal transduction	Signaling by NOTCH	108	22 (20.6%)	2.66E-02	9 (7.6%)	0.307
Signal transduction	NRIF signals cell death from the nucleus	15	6 (40.0%)	2.67E-02	3 (18.8%)	0.257
Signal transduction	Degradation of GLI2 by the proteasome	15	6 (40.0%)	2.67E-02	2 (13.3%)	0.358
Signal transduction	GLI3 is processed to GLI3R by the proteasome	15	6 (40.0%)	2.67E-02	2 (13.3%)	0.358
Signal transduction	Beta-catenin independent WNT signaling	97	20 (21.1%)	2.76E-02	9 (9.8%)	0.229
Signal transduction	Signalling by NGF	433	66 (15.5%)	3.20E-02	NA	NA
Signal transduction	Signaling by NOTCH1	74	16 (21.9%)	3.83E-02	5 (6.8%)	0.442
Signal transduction	Degradation of DVL	13	5 (41.7%)	3.98E-02	2 (16.7%)	0.315
Signal transduction	G alpha (12/13) signalling events	90	18 (20.5%)	4.64E-02	8 (9.2%)	0.268

Supplementary table 2: Hierarchically ordered overrepresented pathways (using DEGs) in hepatic MTs after 0.1% DMSO exposure

Cluster	Pathway	Set size	Amount DEGs (%)	q-value	Amount DEGs with |log2FC|>1 (%)	q-value
Metabolism	Metabolism	2035	472 (23.7%)	9.09E-23	263 (13.5%)	8.62e-05
Metabolism	The citric acid (TCA) cycle and respiratory electron transport	171	63 (37.7%)	3.51E-10	29 (16.8%)	0.108
Metabolism	Glucose metabolism	77	36 (47.4%)	9.87E-09	17 (18.9%)	0.136
Metabolism	Respiratory electron transport, ATP synthesis by chemiosmotic coupling, and heat production by uncoupling proteins.	126	45 (36.6%)	8.78E-07	21 (17.1%)	0.142
Metabolism	Gluconeogenesis	33	19 (59.4%)	1.74E-06	7 (20.6%)	0.308
Metabolism	Biological oxidations	229	66 (30.1%)	2.47E-06	37 (16.4%)	0.0799
Metabolism	Metabolism of carbohydrates	288	80 (28.1%)	2.62E-06	43 (16.3%)	0.0554
Metabolism	Metabolism of lipids and lipoproteins	728	165 (23.0%)	2.87E-06	NA	NA
Metabolism	Respiratory electron transport	103	37 (37.0%)	6.62E-06	18 (18.0%)	0.141
Metabolism	Glycolysis	33	18 (56.2%)	6.85E-06	13 (18.6%)	0.206
Metabolism	Lipoprotein metabolism	68	26 (39.4%)	8.69E-05	NA	NA
Metabolism	Lipid digestion, mobilization, and transport	110	35 (32.4%)	2.92E-04	NA	NA
Metabolism	Pyruvate metabolism and Citric Acid (TCA) cycle	49	20 (41.7%)	3.99E-04	8 (14.8%)	0.552
Metabolism	Phase II conjugation	112	33 (31.1%)	1.03E-03	NA	NA
Metabolism	Activation of gene expression by SREBF (SREBP)	26	13 (50.0%)	1.06E-03	5 (19.2%)	0.444
Metabolism	Fatty acid, triacylglycerol, and ketone body metabolism	153	42 (28.4%)	1.12E-03	NA	NA
Metabolism	Regulation of cholesterol biosynthesis by SREBP (SREBF)	31	14 (45.2%)	1.95E-03	5 (16.1%)	0.556
Metabolism	Citric acid cycle (TCA cycle)	19	10 (52.6%)	3.74E-03	2 (9.1%)	0.859
Metabolism	Sulfur amino acid metabolism	27	12 (46.2%)	3.94E-03	6 (23.1%)	0.27
Metabolism	Phase 1 - Functionalization of compounds	110	31 (29.2%)	4.29E-03	NA	NA
Metabolism	Complex I biogenesis	57	19 (34.5%)	6.10E-03	13 (23.6%)	0.0855
Metabolism	Glucuronidation	24	11 (45.8%)	6.25E-03	7 (28.0%)	0.136
Metabolism	Histidine, lysine, phenylalanine, tyrosine, proline and tryptophan catabolism	45	16 (36.4%)	7.79E-03	11 (23.9%)	0.109
Metabolism	Metabolism of ingested SeMet, Sec, MeSec into H2Se	9	6 (66.7%)	9.88E-03	3 (33.3%)	0.313
Metabolism	Recycling of bile acids and salts	16	8 (50.0%)	1.47E-02	4 (25.0%)	0.37
Metabolism	Pyruvate metabolism	28	11 (40.7%)	1.54E-02	7 (23.3%)	0.222
Metabolism	Regulation of insulin secretion	70	21 (30.0%)	1.68E-02	13 (18.6%)	0.206
Metabolism	Xenobiotics	27	10 (41.7%)	1.97E-02	6 (25.0%)	0.224
Metabolism	Metabolism of polyamines	40	14 (35.0%)	1.97E-02	11 (27.5%)	0.0656
Metabolism	Formation of ATP by chemiosmotic coupling	18	8 (44.4%)	3.02E-02	3 (16.7%)	0.612
Metabolism	Metabolism of vitamins and cofactors	164	38 (23.9%)	3.14E-02	26 (14.7%)	0.249
Metabolism	Mitochondrial Fatty Acid Beta-Oxidation	22	9 (40.9%)	3.21E-02	4 (10.8%)	0.783
Metabolism	Formation of the active cofactor, UDP-glucuronate	3	3 (100.0%)	3.21E-02	2 (66.7%)	0.222
Metabolism	Cytochrome P450 - arranged by substrate type	70	19 (28.8%)	3.61E-02	12 (18.2%)	0.239
Metabolism	Synthesis of very long-chain fatty acyl-CoAs	28	10 (37.0%)	4.16E-02	5 (20.8%)	0.408
Metabolism	Ethanol oxidation	12	6 (50.0%)	4.26E-02	2 (16.7%)	0.676
Vesicle-mediated transport	Vesicle-mediated transport	619	164 (26.8%)	4.81E-11	96 (15.5%)	0.00258
Vesicle-mediated transport	Membrane Trafficking	580	146 (25.4%)	3.18E-08	86 (14.8%)	0.0178
Vesicle-mediated transport	ER to Golgi Anterograde Transport	134	44 (33.6%)	1.01E-05	25 (18.2%)	0.0847
Vesicle-mediated transport	Binding and Uptake of Ligands by Scavenger Receptors	42	20 (50.0%)	1.46E-05	11 (26.8%)	0.0709

(table continued)<br>Cluster	Pathway	Set size	Amount DEGs (%)	q-value	Amount DEGs with |log2FC|>1 (%)	q-value
Vesicle-mediated transport	Scavenging by Class A Receptors	21	12 (63.2%)	1.16E-04	8 (42.1%)	0.0226
Vesicle-mediated transport	Golgi Associated Vesicle Biogenesis	57	22 (39.3%)	4.38E-04	12 (21.4%)	0.136
Vesicle-mediated transport	Clathrin derived vesicle budding	73	26 (36.1%)	4.38E-04	15 (20.8%)	0.109
Vesicle-mediated transport	trans-Golgi Network Vesicle Budding	73	26 (36.1%)	4.38E-04	15 (20.8%)	0.109
Vesicle-mediated transport	COPII (Coat Protein 2) Mediated Vesicle Transport	70	24 (34.8%)	1.47E-03	NA	NA
Vesicle-mediated transport	COPI-mediated anterograde transport	79	25 (32.5%)	3.29E-03	17 (20.5%)	0.0968
Vesicle-mediated transport	Cargo concentration in the ER	34	13 (38.2%)	1.26E-02	6 (18.2%)	0.444
Vesicle-mediated transport	Scavenging by Class B Receptors	5	4 (80.0%)	2.40E-02	3 (50.0%)	0.168
Vesicle-mediated transport	Clathrin-mediated endocytosis	138	34 (24.8%)	2.74E-02	22 (15.9%)	0.201
Vesicle-mediated transport	Lysosome Vesicle Biogenesis	36	12 (34.3%)	3.89E-02	7 (20.0%)	0.33
Extracellular matrix organization	Extracellular matrix organization	295	81 (28.0%)	2.47E-06	57 (19.5%)	0.000318
Extracellular matrix organization	Laminin interactions	25	15 (65.2%)	5.79E-06	9 (39.1%)	0.0194
Extracellular matrix organization	Non-integrin membrane-ECM interactions	46	19 (45.2%)	1.61E-04	10 (23.8%)	0.135
Extracellular matrix organization	Integrin cell surface interactions	68	24 (35.8%)	9.85E-04	14 (21.2%)	0.112
Extracellular matrix organization	Collagen formation	94	26 (28.3%)	1.46E-02	20 (22.0%)	0.0457
Extracellular matrix organization	Collagen biosynthesis and modifying enzymes	70	21 (30.4%)	1.47E-02	18 (26.9%)	0.00951
Disease	Disease	514	124 (24.5%)	3.58E-06	78 (15.4%)	0.0108
Disease	Diseases of signal transduction	251	68 (27.5%)	3.12E-05	40 (16.2%)	0.0703
Disease	Oncogenic MAPK signaling	64	21 (33.9%)	4.64E-03	12 (19.7%)	0.179
Disease	Signaling by BRAF and RAF fusions	61	20 (33.9%)	5.84E-03	11 (19.0%)	0.224
Disease	Hh mutants that don,t undergo autocatalytic processing are degraded by ERAD	12	7 (63.6%)	6.10E-03	2 (18.2%)	0.663
Disease	Hh mutants abrogate ligand secretion	12	7 (63.6%)	6.10E-03	2 (18.2%)	0.663
Disease	Constitutive Signaling by AKT1 E17K in Cancer	25	11 (44.0%)	8.59E-03	8 (32.0%)	0.0802
Disease	Signaling by moderate kinase activity BRAF mutants	39	14 (36.8%)	1.26E-02	7 (18.4%)	0.399
Disease	Paradoxical activation of RAF signaling by kinase inactive BRAF	39	14 (36.8%)	1.26E-02	7 (18.4%)	0.399
Disease	Signaling by high-kinase activity BRAF mutants	35	13 (38.2%)	1.26E-02	6 (17.6%)	0.468
Disease	Defective CFTR causes cystic fibrosis	16	8 (50.0%)	1.47E-02	2 (12.5%)	0.78
Disease	Signaling by RAS mutants	37	13 (36.1%)	2.04E-02	6 (16.7%)	0.52
Disease	ABC transporter disorders	17	8 (47.1%)	2.12E-02	2 (11.8%)	0.783
Disease	Disorders of transmembrane transporters	17	8 (47.1%)	2.12E-02	2 (11.8%)	0.783
Disease	Influenza Infection	63	19 (30.2%)	2.38E-02	12 (19.0%)	0.206
Disease	Nef-mediates down modulation of cell surface receptors by recruiting them to clathrin adapters	22	9 (42.9%)	2.40E-02	7 (31.8%)	0.108
Disease	Host Interactions of HIV factors	91	24 (27.3%)	2.96E-02	16 (18.0%)	0.168
Disease	Vpr-mediated induction of apoptosis by mitochondrial outer membrane permeabilization	3	3 (100.0%)	3.21E-02	2 (66.7%)	0.222
Disease	Infectious disease	254	55 (22.0%)	3.25E-02	37 (14.7%)	0.152
Disease	Listeria monocytogenes entry into host cells	19	8 (42.1%)	4.01E-02	4 (21.1%)	0.453
Disease	Influenza Life Cycle	53	16 (30.2%)	4.12E-02	10 (18.9%)	0.257
Disease	Nef Mediated CD4 Down-regulation	10	5 (55.6%)	4.93E-02	4 (44.4%)	0.136
Immune System	Immune System	1950	371 (19.5%)	1.03E-05	206 (11.3%)	0.26
Immune system	Neutrophil degranulation	497	135 (27.9%)	2.40E-10	76 (15.7%)	0.00844
Immune system	Innate Immune System	1309	275 (21.6%)	1.16E-07	131 (12.4%)	0.136

(table continued)<br>Cluster	Pathway	Set size	Amount DEGs (%)	q-value	Amount DEGs with |log2FC|>1 (%)	q-value
Immune System	Antigen Presentation: Folding, assembly and peptide loading of class I MHC	24	15 (62.5%)	1.03E-05	7 (29.2%)	0.135
Immune System	AKT phosphorylates targets in the cytosol	13	8 (61.5%)	3.84E-03	5 (35.7%)	0.136
Immune System	Regulation of TLR by endogenous ligand	16	9 (56.2%)	3.84E-03	6 (37.5%)	0.0968
Immune System	Terminal pathway of complement	9	6 (75.0%)	4.92E-03	4 (50.0%)	0.109
Immune System	LDL-mediated lipid transport	23	10 (47.6%)	7.33E-03	NA	NA
Immune System	MHC class II antigen presentation	60	19 (32.2%)	1.26E-02	9 (15.3%)	0.498
Immune System	MAP2K and MAPK activation	39	14 (36.8%)	1.26E-02	7 (18.4%)	0.399
Immune System	Regulation of Complement cascade	27	11 (40.7%)	1.54E-02	11 (8.7%)	0.902
Immune System	Antigen processing-Cross presentation	49	16 (32.7%)	2.12E-02	12 (24.5%)	0.0857
Immune System	RAF activation	25	10 (40.0%)	2.57E-02	8 (32.0%)	0.0802
Immune System	Negative regulation of MAPK pathway	40	13 (32.5%)	4.39E-02	7 (17.5%)	0.433
Muscle contraction	Muscle contraction	198	58 (29.7%)	1.41E-05	41 (21.0%)	0.00108
Muscle contraction	Smooth Muscle Contraction	36	20 (57.1%)	1.74E-06	9 (25.7%)	0.126
Muscle contraction	Striated Muscle Contraction	35	19 (54.3%)	6.85E-06	17 (48.6%)	1.29e-05
Metabolism of proteins	Metabolism of proteins	1506	291 (19.7%)	9.34E-05	237 (11.9%)	0.094
Metabolism of proteins	Asparagine N-linked glycosylation	283	83 (29.7%)	1.16E-07	42 (14.7%)	0.136
Metabolism of proteins	Transport to the Golgi and subsequent modification	165	52 (32.1%)	5.53E-06	30 (17.9%)	0.0656
Metabolism of proteins	ER to Golgi Anterograde Transport	134	44 (33.6%)	1.01E-05	25 (18.2%)	0.0847
Metabolism of proteins	Post-translational protein modification	1026	204 (20.2%)	6.00E-04	169 (12.3%)	0.0971
Metabolism of proteins	COPII (Coat Protein 2) Mediated Vesicle Transport	70	24 (34.8%)	1.47E-03	NA	NA
Metabolism of proteins	COPI-mediated anterograde transport	79	25 (32.5%)	3.29E-03	17 (20.5%)	0.0968
Metabolism of proteins	N-glycan trimming in the ER and Calnexin/Calreticulin cycle	34	13 (39.4%)	1.00E-02	3 (9.1%)	0.859
Metabolism of proteins	Cargo concentration in the ER	34	13 (38.2%)	1.26E-02	6 (18.2%)	0.444
Metabolism of proteins	Progressive trimming of alpha-1,2-linked mannose residues from Man9/8/7GlcNAc2 to produce Man5GlcNAc2	3	3 (100.0%)	3.21E-02	2 (66.7%)	0.222
Metabolism of proteins	Synthesis of active ubiquitin: roles of E1 and E2 enzymes	31	11 (36.7%)	3.25E-02	5 (16.7%)	0.552
Cellular responses to stress	Cellular responses to stress	393	88 (22.7%)	2.06E-03	49 (14.3%)	0.136
Cellular responses to stress	LDL-mediated lipid transport	23	10 (47.6%)	7.33E-03	NA	NA
Cellular responses to stress	Detoxification of Reactive Oxygen Species	36	12 (34.3%)	3.89E-02	6 (17.1%)	0.494
Hemostasis	Hemostasis	693	141 (20.6%)	3.22E-03	88 (13.2%)	0.109
Hemostasis	Platelet degranulation	133	56 (42.7%)	4.05E-11	29 (22.5%)	0.0043
Hemostasis	Response to elevated platelet cytosolic Ca2+	138	56 (41.2%)	1.35E-10	29 (21.6%)	0.00819
Hemostasis	Platelet activation, signaling and aggregation	283	78 (27.8%)	5.48E-06	40 (15.4%)	0.109
Hemostasis	Dissolution of Fibrin Clot	13	7 (53.8%)	1.65E-02	3 (23.1%)	0.494
Hemostasis	Ion homeostasis	56	17 (31.5%)	2.38E-02	11 (21.2%)	0.156
Hemostasis	Formation of Fibrin Clot (Clotting Cascade)	39	13 (33.3%)	3.67E-02	10 (25.6%)	0.108
Developmental Biology	Developmental Biology	748	149 (20.2%)	4.90E-03	76 (12.3%)	0.27
Developmental biology	Axon guidance	487	116 (24.1%)	1.46E-05	58 (16.3%)	0.0167
Developmental biology	Semaphorin interactions	68	26 (38.8%)	1.14E-04	14 (21.9%)	0.106
Developmental biology	Sema4D in semaphorin signaling	27	13 (48.1%)	1.60E-03	6 (25.0%)	0.224
Developmental biology	Sema4D induced cell migration and growth-cone collapse	24	12 (50.0%)	1.88E-03	5 (25.0%)	0.285
Developmental biology	EPH-Ephrin signaling	78	24 (30.8%)	7.29E-03	11 (14.9%)	0.473
Developmental biology	MAP2K and MAPK activation	39	14 (36.8%)	1.26E-02	7 (18.4%)	0.399
Developmental Biology	L1CAM interactions	104	28 (27.5%)	1.53E-02	16 (15.7%)	0.308

(table continued)<br>Cluster	Pathway	Set size	Amount DEGs (%)	q-value	Amount DEGs with |log2FC|>1 (%)	q-value
Developmental Biology	Signaling by Robo receptor	33	12 (37.5%)	2.06E-02	NA	NA
Developmental Biology	RAF activation	25	10 (40.0%)	2.57E-02	8 (32.0%)	0.0802
Developmental Biology	Ephrin signaling	19	8 (42.1%)	4.01E-02	5 (26.3%)	0.253
Developmental Biology	Negative regulation of MAPK pathway	40	13 (32.5%)	4.39E-02	7 (17.5%)	0.433
Cell-Cell-communication	Cell-Cell communication	131	35 (27.1%)	6.83E-03	22 (17.9%)	0.113
Cell-Cell-communication	Cell-extracellular matrix interactions	16	10 (62.5%)	7.43E-04	7 (43.8%)	0.0392
Cell-Cell-communication	Cell junction organization	89	27 (31.0%)	3.84E-03	18 (20.5%)	0.0855
Programmed Cell Death	Programmed Cell Death	125	33 (26.8%)	1.04E-02	15 (12.4%)	0.61
Programmed Cell Death	Apoptosis	122	33 (27.5%)	7.33E-03	15 (12.7%)	0.588
Programmed Cell Death	Apoptotic execution phase	53	17 (32.7%)	1.65E-02	7 (13.5%)	0.612
Programmed Cell Death	Apoptotic cleavage of cellular proteins	39	13 (34.2%)	3.08E-02	6 (15.8%)	0.552
Programmed Cell Death	Activation of BAD and translocation to mitochondria	15	7 (46.7%)	3.61E-02	2 (13.3%)	0.76
Programmed Cell Death	Caspase-mediated cleavage of cytoskeletal proteins	13	6 (50.0%)	4.26E-02	4 (33.3%)	0.222
Transport of small molecules	(Transmembrane) transport of small molecules	628	114 (18.4%)	1.23E-01	NA	NA
Transport of small molecules	VLDL interactions	23	11 (47.8%)	4.64E-03	NA	NA
Transport of small molecules	VLDL biosynthesis	5	4 (80.0%)	2.40E-02	NA	NA
Transport of small molecules	ABC-family proteins mediated transport	62	18 (29.5%)	3.44E-02	8 (13.8%)	0.598
Transport of small molecules	Iron uptake and transport	44	14 (32.6%)	3.44E-02	8 (13.8%)	0.598
Transport of small molecules	VLDLR internalisation and degradation	12	6 (50.0%)	4.26E-02	4 (33.3%)	0.222
Signal transduction	Signal transduction	2538	377 (15.1%)	8.50E-01	275 (10.5%)	0.614
Signal transduction	Signaling by Rho GTPases	434	97 (22.8%)	1.03E-03	63 (14.7%)	0.0656
Signal transduction	Signaling by PDGF	331	78 (23.9%)	1.09E-03	18 (34.0%)	0.000678
Signal transduction	RHO GTPases activate PAKs	23	12 (52.2%)	1.16E-03	5 (23.8%)	0.313
Signal transduction	RHO GTPase Effectors	299	70 (23.7%)	2.70E-03	43 (14.4%)	0.142
Signal transduction	PKB-mediated events	41	16 (40.0%)	3.33E-03	1 (50.0%)	0.552
Signal transduction	Signalling by NGF	433	94 (22.1%)	3.54E-03	NA	NA
Signal transduction	VEGFA-VEGFR2 Pathway	282	66 (23.7%)	3.74E-03	18 (19.6%)	0.108
Signal transduction	AKT phosphorylates targets in the cytosol	13	8 (61.5%)	3.84E-03	5 (35.7%)	0.136
Signal transduction	Signaling by MET	63	21 (34.4%)	3.84E-03	12 (19.7%)	0.179
Signal transduction	Signaling by VEGF	290	67 (23.4%)	4.51E-03	19 (19.0%)	0.109
Signal transduction	Signaling by Insulin receptor	278	64 (23.5%)	5.04E-03	12 (16.0%)	0.374
Signal transduction	Signaling by EGFR	319	72 (22.9%)	5.39E-03	10 (23.3%)	0.136
Signal transduction	MET promotes cell motility	29	12 (44.4%)	5.39E-03	5 (18.5%)	0.486
Signal transduction	IRS-related events triggered by IGF1R	254	59 (23.7%)	6.25E-03	11 (22.4%)	0.136
Signal transduction	IGF1R signaling cascade	254	59 (23.7%)	6.25E-03	11 (22.0%)	0.136
Signal transduction	mTOR signalling	40	15 (38.5%)	6.25E-03	13 (32.5%)	0.0103
Signal transduction	Signaling by Type 1 Insulin-like Growth Factor 1 Receptor (IGF1R)	255	59 (23.6%)	6.83E-03	11 (21.6%)	0.142
Signal transduction	NGF signalling via TRKA from the plasma membrane	340	75 (22.3%)	7.33E-03	NA	NA
Signal transduction	PI3K Cascade	80	24 (30.4%)	8.48E-03	6 (14.6%)	0.599
Signal transduction	MET activates PTK2 signaling	20	9 (50.0%)	8.59E-03	4 (22.2%)	0.426
Signal transduction	RHO GTPases activate PKNs	96	27 (28.7%)	1.01E-02	17 (17.9%)	0.161
Signal transduction	IRS-mediated signalling	250	57 (23.3%)	1.04E-02	9 (20.0%)	0.249
Signal transduction	MAP2K and MAPK activation	39	14 (36.8%)	1.26E-02	7 (18.4%)	0.399
Signal transduction	Insulin receptor signalling cascade	253	57 (23.0%)	1.33E-02	9 (17.6%)	0.369

(table continued) Cluster	Pathway	Set size	Amount DEGs (%)	q-value	Amount DEGs with |log2FC|>1 (%)	q-value
Signal transduction	RHO GTPases activate IQGAPs	13	7 (53.8%)	1.65E-02	1 (9.1%)	0.868
Signal transduction	RHO GTPases Activate ROCKs	17	8 (47.1%)	2.12E-02	6 (31.6%)	0.136
Signal transduction	RAF activation	25	10 (40.0%)	2.57E-02	8 (32.0%)	0.0802
Signal transduction	VEGFR2 mediated vascular permeability	29	11 (37.9%)	2.60E-02	6 (22.2%)	0.302
Signal transduction	Downregulation of ERBB4 signaling	8	5 (62.5%)	3.02E-02	2 (25.0%)	0.552
Signal transduction	Downstream signal transduction	307	65 (21.5%)	3.02E-02	7 (24.1%)	0.206
Signal transduction	Negative regulation of MAPK pathway	40	13 (32.5%)	4.39E-02	7 (17.5%)	0.433
Signal transduction	GAB1 signalosome	128	31 (24.2%)	4.93E-02	1 (9.1%)	0.868
Signal transduction	SHC-related events triggered by IGF1R	9	5 (55.6%)	4.93E-02	5 (55.6%)	0.0504
Neuronal system	Neuronal system	351	44 (12.6%)	9.84E-01	31 (8.4%)	0.957
Neuronal system	Chylomicron-mediated lipid transport	23	12 (54.5%)	7.48E-04	NA	NA
Gene expression	Gene expression	1755	239 (14.0%)	1.00E+00	NA	NA
Gene expression	TP53 Regulates Metabolic Genes	86	25 (29.4%)	1.04E-02	12 (14.0%)	0.53

Supplementary table 3: Hierarchically ordered overrepresented pathways (using DEPs) in cardiac MTs after 0.1% DMSO exposure

Cluster	Pathway	Set size	Amount DEGs (%)	q-value
Cellular responses to stress	Cellular Senescence	192	38 (20.2%)	5.85E-07
Cellular responses to stress	Cellular responses to stress	393	70 (18.1%)	4.67E-10
Cellular responses to stress	Senescence-Associated Secretory Phenotype (SASP)	113	32 (28.8%)	8.03E-10
Cellular responses to stress	Oxidative Stress Induced Senescence	129	34 (27.0%)	1.37E-09
Cellular responses to stress	Detoxification of Reactive Oxygen Species	36	15 (42.9%)	2.01E-07
Cellular responses to stress	Cellular response to heat stress	100	15 (15.2%)	3.82E-02
Disease	Disease	514	58 (11.6%)	9.06E-03
Disease	Deregulated CDK5 triggers multiple neurodegenerative pathways in Alzheimer,s disease models	18	8 (44.4%)	2.17E-04
Disease	Neurodegenerative Diseases	18	8 (44.4%)	2.17E-04
Disease	Signaling by RAS mutants	37	10 (27.8%)	1.93E-03
Disease	Signaling by moderate kinase activity BRAF mutants	39	10 (26.3%)	2.94E-03
Disease	Paradoxical activation of RAF signaling by kinase inactive BRAF	39	10 (26.3%)	2.94E-03
Disease	Signaling by high-kinase activity BRAF mutants	35	9 (26.5%)	5.15E-03
Disease	Oncogenic MAPK signaling	64	13 (20.6%)	5.58E-03
Disease	Host Interactions of HIV factors	91	16 (18.4%)	5.82E-03
Disease	Signaling by BRAF and RAF fusions	61	12 (20.0%)	1.01E-02
Disease	Integration of provirus	10	4 (44.4%)	1.45E-02
Disease	APOBEC3G mediated resistance to HIV-1 infection	7	3 (50.0%)	3.00E-02
Disease	Infectious disease	254	30 (12.2%)	4.00E-02
Disease	Uptake and function of anthrax toxins	12	4 (33.3%)	4.00E-02
Disease	2-LTR circle formation	7	3 (42.9%)	4.64E-02
Vesicle-mediated transport	Vesicle-mediated transport	619	82 (13.4%)	9.87E-06
Vesicle-mediated transport	COPI-mediated anterograde transport	79	20 (26.0%)	1.22E-05
Vesicle-mediated transport	ER to Golgi Anterograde Transport	134	27 (20.8%)	2.17E-05
Vesicle-mediated transport	Membrane Trafficking	580	74 (12.9%)	1.15E-04
Vesicle-mediated transport	Translocation of GLUT4 to the plasma membrane	34	11 (32.4%)	2.52E-04
Vesicle-mediated transport	Golgi-to-ER retrograde transport	111	20 (18.2%)	1.94E-03
Vesicle-mediated transport	Clathrin-mediated endocytosis	138	22 (16.1%)	5.45E-03
Vesicle-mediated transport	COPI-independent Golgi-to-ER retrograde traffic	28	8 (28.6%)	5.58E-03
Vesicle-mediated transport	COPII (Coat Protein 2) Mediated Vesicle Transport	70	13 (19.1%)	1.01E-02
Vesicle-mediated transport	Gap junction trafficking and regulation	14	5 (35.7%)	1.40E-02
Vesicle-mediated transport	Formation of annular gap junctions	9	4 (44.4%)	1.45E-02
Vesicle-mediated transport	Gap junction degradation	10	4 (40.0%)	2.14E-02
Vesicle-mediated transport	Gap junction trafficking	12	4 (33.3%)	4.00E-02
Vesicle-mediated transport	Binding and Uptake of Ligands by Scavenger Receptors	42	8 (20.0%)	4.06E-02
Metabolism of proteins	Metabolism of proteins	1506	259 (17.6%)	6.70E-38
Metabolism of proteins	Translation	177	86 (53.1%)	1.45E-49
Metabolism of proteins	L13a-mediated translational silencing of Ceruloplasmin expression	129	73 (63.5%)	1.45E-49
Metabolism of proteins	3, -UTR-mediated translational regulation	129	73 (63.5%)	1.45E-49
Metabolism of proteins	GTP hydrolysis and joining of the 60S ribosomal subunit	129	73 (62.9%)	2.72E-49
Metabolism of proteins	Cap-dependent Translation Initiation	137	74 (60.2%)	4.09E-48

(table continued) Cluster	Pathway	Set size	Amount DEGs (%)	q-value
Metabolism of proteins	Eukaryotic Translation Initiation	137	74 (60.2%)	4.09E-48
Metabolism of proteins	Eukaryotic Translation Elongation	108	65 (67.0%)	1.40E-46
Metabolism of proteins	Eukaryotic Translation Termination	106	62 (65.3%)	1.76E-43
Metabolism of proteins	Formation of a pool of free 40S subunits	117	65 (61.9%)	1.76E-43
Metabolism of proteins	Peptide chain elongation	103	61 (66.3%)	1.93E-43
Metabolism of proteins	SRP-dependent cotranslational protein targeting to membrane	126	65 (57.0%)	1.93E-40
Metabolism of proteins	Ribosomal scanning and start codon recognition	69	39 (62.9%)	1.96E-26
Metabolism of proteins	Translation initiation complex formation	70	39 (62.9%)	1.96E-26
Metabolism of proteins	Activation of the mRNA upon binding of the cap-binding complex and eIFs, and subsequent binding to 43S	71	39 (61.9%)	4.57E-26
Metabolism of proteins	Formation of the ternary complex, and subsequently, the 43S complex	61	32 (58.2%)	3.42E-20
Metabolism of proteins	Amyloid fiber formation	83	32 (39.0%)	8.38E-14
Metabolism of proteins	Cytosolic tRNA aminoacylation	26	17 (70.8%)	6.14E-13
Metabolism of proteins	tRNA Aminoacylation	45	19 (45.2%)	1.04E-09
Metabolism of proteins	Formation of tubulin folding intermediates by CCT/TriC	26	15 (57.7%)	1.22E-09
Metabolism of proteins	UCH proteinases	107	30 (29.1%)	2.17E-09
Metabolism of proteins	Prefoldin mediated transfer of substrate to CCT/TriC	29	15 (53.6%)	4.62E-09
Metabolism of proteins	Cooperation of Prefoldin and TriC/CCT in actin and tubulin folding	34	16 (48.5%)	8.13E-09
Metabolism of proteins	Folding of actin by CCT/TriC	10	8 (80.0%)	5.96E-07
Metabolism of proteins	Mitochondrial protein import	63	19 (30.2%)	2.06E-06
Metabolism of proteins	Protein folding	106	23 (22.3%)	3.21E-05
Metabolism of proteins	Chaperonin-mediated protein folding	100	22 (22.7%)	3.88E-05
Metabolism of proteins	Protein methylation	20	8 (40.0%)	5.09E-04
Metabolism of proteins	Ub-specific processing proteases	225	34 (15.3%)	8.68E-04
Metabolism of proteins	Transport to the Golgi and subsequent modification	165	27 (16.8%)	8.91E-04
Metabolism of proteins	Association of TriC/CCT with target proteins during biosynthesis	40	11 (28.2%)	9.08E-04
Metabolism of proteins	Post-chaperonin tubulin folding pathway	23	8 (34.8%)	1.44E-03
Metabolism of proteins	Asparagine N-linked glycosylation	283	39 (14.0%)	1.75E-03
Metabolism of proteins	Cooperation of PDCL (PhLP1) and TRiC/CCT in G-protein beta folding	44	11 (25.6%)	2.14E-03
Metabolism of proteins	Deubiquitination	302	38 (12.8%)	9.06E-03
Chromatin organization	Chromatin organization	274	34 (12.6%)	1.79E-02
Chromatin organization	HDMs demethylate histones	52	27 (52.9%)	1.31E-15
Chromatin organization	RMTs methylate histone arginines	74	32 (43.2%)	2.98E-15
Chromatin organization	PKMTs methylate histone lysines	73	28 (39.4%)	2.88E-12
Chromatin organization	HDACs deacetylate histones	94	29 (30.9%)	1.03E-09
Chromatin organization	HATs acetylate histones	143	29 (20.4%)	1.39E-05
Chromatin organization	Chromatin modifying enzymes	274	34 (12.6%)	1.79E-02
Muscle contraction	Muscle contraction	198	32 (16.5%)	3.42E-04
Muscle contraction	Striated Muscle Contraction	35	15 (42.9%)	2.01E-07
Muscle contraction	Smooth Muscle Contraction	36	13 (37.1%)	1.08E-05
Gene expression	Gene Expression	1755	233 (13.7%)	9.34E-18
Gene expression	Nonsense Mediated Decay (NMD) independent of the Exon Junction Complex (EJC)	109	63 (64.9%)	7.07E-44
Gene expression	Nonsense Mediated Decay (NMD) enhanced by the Exon Junction Complex (EJC)	122	64 (58.7%)	7.04E-41

(table continued) Cluster	Pathway	Set size	Amount DEGs (%)	q-value
Gene expression	Nonsense-Mediated Decay (NMD)	122	64 (58.7%)	7.04E-41
Gene expression	ERCC6 (CSB) and EHMT2 (G9a) positively regulate rRNA expression	79	33 (42.3%)	2.35E-15
Gene expression	RNA Polymerase I Promoter Opening	66	30 (46.2%)	2.98E-15
Gene expression	DNA methylation	68	30 (44.8%)	7.63E-15
Gene expression	SIRT1 negatively regulates rRNA Expression	72	30 (42.9%)	3.09E-14
Gene expression	PRC2 methylates histones and DNA	77	31 (41.3%)	3.49E-14
Gene expression	Positive epigenetic regulation of rRNA expression	111	34 (31.5%)	1.54E-11
Gene expression	RNA Polymerase I Chain Elongation	94	31 (33.7%)	1.99E-11
Gene expression	B-WICH complex positively regulates rRNA expression	96	31 (33.3%)	2.63E-11
Gene expression	RNA Polymerase I Transcription	115	34 (30.1%)	5.69E-11
Gene expression	RNA Polymerase I Promoter Clearance	113	32 (28.8%)	8.03E-10
Gene expression	Transcriptional regulation by small RNAs	108	31 (29.0%)	1.31E-09
Gene expression	NoRC negatively regulates rRNA expression	110	31 (28.7%)	1.65E-09
Gene expression	Negative epigenetic regulation of rRNA expression	113	31 (27.9%)	3.42E-09
Gene expression	mRNA Splicing - Major Pathway	185	39 (22.2%)	2.98E-08
Gene expression	RNA Polymerase I, RNA Polymerase III, and Mitochondrial Transcription	153	35 (23.2%)	5.57E-08
Gene expression	Gene Silencing by RNA	134	32 (24.4%)	6.40E-08
Gene expression	mRNA Splicing	193	39 (21.2%)	1.05E-07
Gene expression	Epigenetic regulation of gene expression	154	34 (22.7%)	1.58E-07
Gene expression	Processing of Capped Intron-Containing Pre-mRNA	248	43 (18.1%)	2.17E-06
Gene expression	TP53 Regulates Metabolic Genes	86	21 (24.7%)	1.54E-05
Gene expression	AUF1 (hnRNP D0) binds and destabilizes mRNA	7	5 (83.3%)	1.22E-04
Gene expression	Deadenylation of mRNA	25	7 (30.4%)	7.37E-03
Gene expression	Regulation of mRNA stability by proteins that bind AU-rich elements	40	9 (24.3%)	8.96E-03
Gene expression	mRNA 3,-end processing	64	12 (20.3%)	8.96E-03
Gene expression	Post-Elongation Processing of Intron-Containing pre-mRNA	64	12 (20.3%)	8.96E-03
Gene expression	Cleavage of Growing Transcript in the Termination Region	73	12 (17.6%)	2.45E-02
Gene expression	RNA Polymerase II Transcription Termination	73	12 (17.6%)	2.45E-02
Gene expression	Post-Elongation Processing of the Transcript	73	12 (17.6%)	2.45E-02
Metabolism	Metabolism	2035	311 (15.6%)	6.60E-36
Metabloism	Beta oxidation of lauroyl-CoA to decanoyl-CoA-CoA	5	3 (60.0%)	1.74E-02
Metabolism	Selenoamino acid metabolism	132	69 (58.0%)	1.76E-43
Metabolism	Selenocysteine synthesis	105	61 (64.9%)	1.27E-42
Metabolism	Metabolism of amino acids and derivatives	328	97 (31.3%)	1.65E-32
Metabolism	The citric acid (TCA) cycle and respiratory electron transport	171	70 (41.9%)	2.34E-32
Metabolism	Respiratory electron transport, ATP synthesis by chemiosmotic coupling, and heat production by uncoupling proteins.	126	47 (38.2%)	1.24E-19
Metabolism	Respiratory electron transport	103	37 (37.0%)	5.72E-15
Metabolism	Pyruvate metabolism and Citric Acid (TCA) cycle	49	23 (47.9%)	2.88E-12
Metabolism	Glucose metabolism	77	28 (36.8%)	1.99E-11
Metabolism	Citric acid cycle (TCA cycle)	19	14 (73.7%)	4.25E-11
Metabolism	Formation of ATP by chemiosmotic coupling	18	10 (55.6%)	2.17E-06
Metabolism	Gluconeogenesis	33	13 (40.6%)	3.42E-06

(table continued)<br>Cluster	Pathway	Set size	Amount DEGs (%)	q-value
Metabolism	Branched-chain amino acid catabolism	24	11 (47.8%)	3.60E-06
Metabolism	Complex I biogenesis	57	16 (29.1%)	2.56E-05
Metabolism	Metabolism of carbohydrates	288	44 (15.5%)	7.91E-05
Metabolism	Glycogen breakdown (glycogenolysis)	17	8 (47.1%)	1.33E-04
Metabolism	Mitochondrial Fatty Acid Beta-Oxidation	22	9 (40.9%)	1.57E-04
Metabolism	mitochondrial fatty acid beta-oxidation of saturated fatty acids	10	6 (60.0%)	2.57E-04
Metabolism	SeMet incorporation into proteins	13	6 (54.5%)	5.09E-04
Metabolism	Glycolysis	33	10 (31.2%)	7.34E-04
Metabolism	Synthesis and interconversion of nucleotide di- and triphosphates	27	9 (33.3%)	8.91E-04
Metabolism	Pyruvate metabolism	28	9 (33.3%)	8.91E-04
Metabolism	Beta oxidation of hexanoyl-CoA to butanoyl-CoA	5	4 (80.0%)	1.06E-03
Metabolism	Beta oxidation of decanoyl-CoA to octanoyl-CoA-CoA	5	4 (80.0%)	1.06E-03
Metabolism	Beta oxidation of octanoyl-CoA to hexanoyl-CoA	5	4 (80.0%)	1.06E-03
Metabolism	Glyoxylate metabolism and glycine degradation	25	8 (32.0%)	2.57E-03
Metabolism	Beta oxidation of palmitoyl-CoA to myristoyl-CoA	3	3 (100.0%)	2.66E-03
Metabolism	Beta-oxidation of very long chain fatty acids	6	4 (66.7%)	2.74E-03
Metabolism	Metabolism of nucleotides	100	17 (17.2%)	8.67E-03
Metabolism	Glutathione conjugation	40	9 (23.1%)	1.22E-02
Metabolism	Pentose phosphate pathway (hexose monophosphate shunt)	9	4 (44.4%)	1.45E-02
Metabolism	Keratan sulfate degradation	5	3 (60.0%)	1.74E-02
Metabolism	Glycogen synthesis	10	4 (40.0%)	2.14E-02
Metabolism	Insulin effects increased synthesis of Xylulose-5-Phosphate	2	2 (100.0%)	2.39E-02
Metabolism	Biotin transport and metabolism	11	4 (36.4%)	3.00E-02
Metabolism	Ethanol oxidation	12	4 (33.3%)	4.00E-02
Metabolism	eNOS activation	12	4 (33.3%)	4.00E-02
Metabolism	Purine ribonucleoside monophosphate biosynthesis	12	4 (33.3%)	4.00E-02
Extracellular matrix organization	Extracellular matrix organization	295	43 (15.0%)	2.25E-04
Extracellular matrix organization	Non-integrin membrane-ECM interactions	46	15 (35.7%)	3.23E-06
Extracellular matrix organization	Laminin interactions	25	11 (47.8%)	3.60E-06
Extracellular matrix organization	Syndecan interactions	21	8 (40.0%)	5.09E-04
Extracellular matrix organization	ECM proteoglycans	57	13 (23.6%)	1.57E-03
Extracellular matrix organization	Integrin cell surface interactions	68	13 (19.4%)	9.06E-03
Extracellular matrix organization	Collagen formation	94	14 (15.6%)	3.82E-02
Immune system	Immune System	1950	197 (10.4%)	1.07E-04
Immune system	Neutrophil degranulation	497	111 (22.7%)	6.05E-24
Immune system	Innate Immune System	1309	159 (12.5%)	1.13E-08
Immune system	Interleukin-7 signaling	26	13 (50.0%)	1.85E-07
Immune system	Advanced glycosylation endproduct receptor signaling	13	6 (46.2%)	1.46E-03
Immune system	MAP2K and MAPK activation	39	9 (23.7%)	1.01E-02
Immune system	Antigen Presentation: Folding, assembly and peptide loading of class I MHC	24	6 (25.0%)	3.38E-02
Immune system	Activation of C3 and C5	7	3 (42.9%)	4.64E-02

(table continued) Cluster	Pathway	Set size	Amount DEGs (%)	q-value
Cell Cycle	Cell Cycle	551	71 (13.1%)	1.02E-04
Cell Cycle	Meiotic recombination	65	30 (46.9%)	1.87E-15
Cell Cycle	Condensation of Prophase Chromosomes	77	30 (39.5%)	4.12E-13
Cell Cycle	Meiosis	79	30 (39.0%)	6.11E-13
Cell Cycle	Mitotic Prophase	143	39 (27.5%)	3.80E-11
Cell Cycle	Deposition of new CENPA-containing nucleosomes at the centromere	43	17 (39.5%)	1.05E-07
Cell Cycle	Nucleosome assembly	43	17 (39.5%)	1.05E-07
Cell Cycle	M Phase	267	46 (17.6%)	2.04E-06
Cell Cycle	Meiotic synapsis	48	15 (31.9%)	1.46E-05
Cell Cycle	G2/M DNA damage checkpoint	97	22 (22.9%)	3.27E-05
Cell Cycle	Cell Cycle, Mitotic	468	63 (13.7%)	8.50E-05
Cell Cycle	Packaging Of Telomere Ends	54	15 (27.8%)	8.91E-05
Cell Cycle	Regulation of PLK1 Activity at G2/M Transition	90	19 (21.6%)	3.01E-04
Cell Cycle	Chk1/Chk2(Cds1) mediated inactivation of Cyclin B:Cdk1 complex	12	6 (50.0%)	8.91E-04
Cell Cycle	G2/M Checkpoints	121	22 (18.3%)	9.48E-04
Cell Cycle	Telomere Maintenance	85	16 (19.0%)	4.09E-03
Cell Cycle	Cell Cycle Checkpoints	158	24 (15.4%)	5.95E-03
Cell Cycle	G2/M Transition	136	21 (15.8%)	8.25E-03
Cell Cycle	Mitotic G2-G2/M phases	138	21 (15.6%)	9.28E-03
Cell Cycle	Chromosome Maintenance	113	18 (16.1%)	1.24E-02
Cell Cycle	Loss of Nlp from mitotic centrosomes	72	13 (18.6%)	1.27E-02
Cell Cycle	Loss of proteins required for interphase microtubule organization from the centrosome	72	13 (18.6%)	1.27E-02
Cell Cycle	Golgi Cisternae Pericentriolar Stack Reorganization	14	5 (35.7%)	1.40E-02
Cell Cycle	Recruitment of mitotic centrosome proteins and complexes	82	14 (17.5%)	1.51E-02
Cell Cycle	Centrosome maturation	82	14 (17.5%)	1.51E-02
Cell Cycle	AURKA Activation by TPX2	75	13 (17.8%)	1.74E-02
DNA repair	DNA repair	323	30 (9.5%)	4.02E-01
DNA repair	Nonhomologous End-Joining (NHEJ)	71	18 (25.7%)	4.27E-05
DNA repair	Recruitment and ATM-mediated phosphorylation of repair and signaling proteins at DNA double strand breaks	77	17 (22.1%)	5.36E-04
DNA repair	DNA Double Strand Break Response	78	17 (21.8%)	6.31E-04
DNA repair	Processing of DNA double-strand break ends	100	17 (17.2%)	8.67E-03
DNA repair	DNA Damage Recognition in GG-NER	39	9 (23.7%)	1.01E-02
Organelle biogenesis and maintenance	Organelle biogenesis and maintenance	310	37 (12.2%)	2.14E-02
Organelle biogenesis and maintenance	BBSome-mediated cargo-targeting to cilium	23	6 (26.1%)	2.81E-02
Organelle biogenesis and maintenance	Cilium Assembly	192	24 (12.8%)	4.55E-02
Developmental Biology	Developmental Biology	748	77 (10.4%)	2.45E-02
Developmental Biology	L1CAM interactions	104	19 (18.8%)	1.75E-03
Developmental biology	Axon guidance	487	59 (12.3%)	2.42E-03
Developmental Biology	Signal transduction by L1	22	7 (31.8%)	5.63E-03
Developmental biology	CHL1 interactions	9	4 (44.4%)	1.45E-02
Developmental Biology	Recycling pathway of L1	29	7 (25.0%)	2.01E-02
Hemostasis	Hemostasis	693	87 (13.0%)	1.45E-05

(table continued) Cluster	Pathway	Set size	Amount DEGs (%)	q-value
Hemostasis	Platelet degranulation	133	42 (32.3%)	1.42E-14
Hemostasis	Response to elevated platelet cytosolic Ca2+	138	42 (31.1%)	5.82E-14
Hemostasis	Platelet activation, signaling and aggregation	283	49 (17.6%)	8.19E-07
Hemostasis	Factors involved in megakaryocyte development and platelet production	133	26 (19.8%)	7.45E-05
Hemostasis	Reduction of cytosolic Ca++ levels	14	6 (42.9%)	2.28E-03
Hemostasis	Ion homeostasis	56	10 (18.5%)	3.22E-02
Cell-Cell-communication	Cell-Cell communication	131	19 (14.8%)	2.14E-02
Cell-Cell-communication	Cell-extracellular matrix interactions	16	8 (50.0%)	8.03E-05
Cell-Cell-communication	Cell junction organization	89	14 (16.3%)	2.70E-02
Transport of small molecules	(Transmembrane) transport of small molecules	628	26 (4.2%)	1.00E+00
Transport of small molecules	Sodium/Calcium exchangers	13	5 (38.5%)	1.01E-02
Neuronal System	Neuronal System	351	20 (5.8%)	1.00E+00
Neuronal System	Activation of CaMK IV	4	3 (75.0%)	8.81E-03
Neuronal System	CREB phosphorylation through the activation of CaMKK	6	3 (50.0%)	3.00E-02
Neuronal System	CREB phosphorylation through the activation of CaMKII	17	5 (29.4%)	3.07E-02
Neuronal System	Ras activation uopn Ca2+ infux through NMDA receptor	19	5 (26.3%)	4.76E-02
Signal transduction	Signal transduction	2538	160 (6.4%)	1.00E+00
Signal transduction	RHO GTPases activate PKNs	96	42 (44.2%)	2.17E-20
Signal transduction	RHO GTPase Effectors	299	68 (23.1%)	6.14E-15
Signal transduction	Activated PKN1 stimulates transcription of AR (androgen receptor) regulated genes KLK2 and KLK3	70	30 (43.5%)	1.96E-14
Signal transduction	Formation of the beta-catenin:TCF transactivating complex	95	31 (33.3%)	2.63E-11
Signal transduction	Signaling by Rho GTPases	434	75 (17.7%)	2.78E-10
Signal transduction	RHO GTPases activate PAKs	23	11 (47.8%)	3.60E-06
Signal transduction	Signaling by Wnt	286	46 (16.5%)	1.11E-05
Signal transduction	TCF dependent signaling in response to WNT	195	35 (18.5%)	1.39E-05
Signal transduction	MET promotes cell motility	29	8 (29.6%)	4.38E-03
Signal transduction	RHO GTPases Activate ROCKs	17	6 (35.3%)	6.88E-03
Signal transduction	RHO GTPases Activate WASPs and WAVEs	37	9 (24.3%)	8.96E-03
Signal transduction	MET activates PTK2 signaling	20	6 (33.3%)	9.00E-03
Signal transduction	RHO GTPases activate IQGAPs	13	5 (38.5%)	1.01E-02
Signal transduction	DARPP-32 events	27	7 (26.9%)	1.38E-02
Signal transduction	CaMK IV-mediated phosphorylation of CREB	5	3 (60.0%)	1.74E-02
Signal transduction	Ca-dependent events	30	7 (23.3%)	2.84E-02
Signal transduction	Cam-PDE 1 activation	6	3 (50.0%)	3.00E-02
Programmed Cell Death	Programmed Cell Death	125	27 (22.1%)	6.48E-06
Programmed Cell Death	Apoptotic execution phase	53	17 (32.7%)	2.37E-06
Programmed Cell Death	Apoptosis	122	27 (22.7%)	3.86E-06
Programmed Cell Death	Apoptotic cleavage of cellular proteins	39	11 (28.9%)	7.34E-04
Programmed Cell Death	Activation of BAD and translocation to mitochondria	15	6 (40.0%)	3.39E-03
Programmed Cell Death	Caspase-mediated cleavage of cytoskeletal proteins	13	5 (41.7%)	7.39E-03
Programmed Cell Death	Activation of BH3-only proteins	31	8 (26.7%)	8.67E-03
Programmed Cell Death	Activation of DNA fragmentation factor	13	5 (38.5%)	1.01E-02
Programmed Cell Death	Apoptosis induced DNA fragmentation	13	5 (38.5%)	1.01E-02
Programmed Cell Death	Intrinsic Pathway for Apoptosis	44	9 (20.9%)	2.17E-02

Supplementary table 4: Hierarchically ordered overrepresented pathways (using DEPs) in hepatic MTs after 0.1% DMSO exposure

Cluster	Pathway	Set size	Amount DEGs (%)	q-value
Metabolism	Metabolism	2035	240 (12.1%)	6.62E-43
Metabolism	Metabolism of amino acids and derivatives	328	64 (20.6%)	1.11E-20
Metabolism	Selenoamino acid metabolism	132	38 (31.9%)	8.05E-19
Metabolism	Selenocysteine synthesis	105	29 (30.9%)	3.06E-14
Metabolism	The citric acid (TCA) cycle and respiratory electron transport	171	29 (17.4%)	6.42E-08
Metabolism	Glucose metabolism	77	19 (25.0%)	7.16E-08
Metabolism	Peroxisomal lipid metabolism	29	12 (41.4%)	1.00E-07
Metabolism	Respiratory electron transport, ATP synthesis by chemiosmotic coupling, and heat production by uncoupling proteins.	126	24 (19.5%)	1.29E-07
Metabolism	Metabolism of lipids and lipoproteins	728	68 (9.5%)	2.08E-06
Metabolism	Biological oxidations	229	31 (14.0%)	2.48E-06
Metabolism	Fructose metabolism	7	5 (71.4%)	6.11E-05
Metabolism	Respiratory electron transport	103	17 (17.0%)	7.80E-05
Metabolism	Branched-chain amino acid catabolism	24	8 (34.8%)	8.87E-05
Metabolism	eNOS activation	12	6 (50.0%)	1.02E-04
Metabolism	Metabolism of carbohydrates	288	32 (11.3%)	1.04E-04
Metabolism	Formation of ATP by chemiosmotic coupling	18	7 (38.9%)	1.30E-04
Metabolism	Gluconeogenesis	33	9 (28.1%)	1.57E-04
Metabolism	Phase 1 - Functionalization of compounds	110	17 (15.7%)	1.84E-04
Metabolism	eNOS activation and regulation	23	7 (30.4%)	6.33E-04
Metabolism	Metabolism of nitric oxide	23	7 (30.4%)	6.33E-04
Metabolism	Glycogen breakdown (glycogenolysis)	17	6 (35.3%)	7.85E-04
Metabolism	Cholesterol biosynthesis	24	7 (29.2%)	7.98E-04
Metabolism	Synthesis of bile acids and bile salts via 7alpha-hydroxycholesterol	24	7 (29.2%)	7.98E-04
Metabolism	Citric acid cycle (TCA cycle)	19	6 (31.6%)	1.37E-03
Metabolism	Complex I biogenesis	57	10 (18.2%)	1.86E-03
Metabolism	Metabolism of ingested H2SeO4 and H2SeO3 into H2Se	4	3 (75.0%)	2.60E-03
Metabolism	Regulation of cholesterol biosynthesis by SREBP (SREBF)	31	7 (22.6%)	3.51E-03
Metabolism	Phase II conjugation	112	14 (13.1%)	4.18E-03
Metabolism	Beta-oxidation of pristanoyl-CoA	10	4 (40.0%)	4.98E-03
Metabolism	Metabolism of vitamins and cofactors	164	18 (11.2%)	5.12E-03
Metabolism	Alpha-oxidation of phytanate	5	3 (60.0%)	5.48E-03
Metabolism	Fructose catabolism	5	3 (60.0%)	5.48E-03
Metabolism	Glyoxylate metabolism and glycine degradation	25	6 (24.0%)	5.50E-03
Metabolism	Synthesis of bile acids and bile salts	34	7 (20.6%)	5.63E-03
Metabolism	Histidine, lysine, phenylalanine, tyrosine, proline and tryptophan catabolism	45	8 (18.2%)	5.93E-03
Metabolism	SeMet incorporation into proteins	13	4 (36.4%)	6.93E-03
Metabolism	Phenylalanine and tyrosine catabolism	11	4 (36.4%)	6.93E-03
Metabolism	Synthesis and interconversion of nucleotide di- and triphosphates	27	6 (22.2%)	7.91E-03
Metabolism	Metabolism of porphyrins	19	5 (26.3%)	8.55E-03
Metabolism	Ethanol oxidation	12	4 (33.3%)	9.01E-03

(table continued) Cluster	Pathway	Set size	Amount DEGs (%)	q-value
Metabolism	Tetrahydrobiopterin (BH4) synthesis, recycling, salvage and regulation	12	4 (33.3%)	9.01E-03
Metabolism	Beta-oxidation of very long chain fatty acids	6	3 (50.0%)	9.01E-03
Metabolism	Biogenic amines are oxidatively deaminated to aldehydes by MAOA and MAOB	2	2 (100.0%)	9.76E-03
Metabolism	Fructose biosynthesis	2	2 (100.0%)	9.76E-03
Metabolism	Synthesis of bile acids and bile salts via 24-hydroxycholesterol	14	4 (28.6%)	1.46E-02
Metabolism	Glycolysis	33	6 (18.8%)	1.57E-02
Metabolism	Bile acid and bile salt metabolism	43	7 (16.3%)	1.71E-02
Metabolism	Lipoprotein metabolism	68	9 (13.6%)	1.76E-02
Metabolism	Synthesis of Ketone Bodies	8	3 (37.5%)	1.93E-02
Metabolism	Heme degradation	8	3 (37.5%)	1.93E-02
Metabolism	Fatty acid, triacylglycerol, and ketone body metabolism	153	15 (10.1%)	2.20E-02
Metabolism	Utilization of Ketone Bodies	3	2 (66.7%)	2.30E-02
Metabolism	Electron transport from NADPH to Ferredoxin	3	2 (66.7%)	2.30E-02
Metabolism	Beta oxidation of palmitoyl-CoA to myristoyl-CoA	3	2 (66.7%)	2.30E-02
Metabolism	Beta oxidation of myristoyl-CoA to lauroyl-CoA	3	2 (66.7%)	2.30E-02
Metabolism	Activation of gene expression by SREBF (SREBP)	26	5 (19.2%)	2.54E-02
Metabolism	Sulfur amino acid metabolism	27	5 (19.2%)	2.54E-02
Metabolism	Ketone body metabolism	9	3 (33.3%)	2.54E-02
Metabolism	Mitochondrial iron-sulfur cluster biogenesis	9	3 (33.3%)	2.54E-02
Metabolism	Pentose phosphate pathway (hexose monophosphate shunt)	9	3 (33.3%)	2.54E-02
Metabolism	Metabolism of water-soluble vitamins and cofactors	113	12 (10.8%)	2.71E-02
Metabolism	Pyruvate metabolism and Citric Acid (TCA) cycle	49	7 (14.6%)	2.73E-02
Metabolism	Metabolism of nucleotides	100	11 (11.1%)	2.96E-02
Metabolism	Pregnenolone biosynthesis	10	3 (30.0%)	3.35E-02
Metabolism	Vitamins B6 activation to pyridoxal phosphate	4	2 (50.0%)	4.01E-02
Metabolism	Amine Oxidase reactions	4	2 (50.0%)	4.01E-02
Metabolism	Nicotinamide salvaging	11	3 (27.3%)	4.22E-02
Metabolism	Retinoid metabolism and transport	44	6 (14.0%)	4.99E-02
Vesicle-mediated transport	Vesicle-mediated transport	619	52 (8.5%)	6.33E-04
Vesicle-mediated transport	Translocation of GLUT4 to the plasma membrane	34	10 (29.4%)	4.01E-05
Vesicle-mediated transport	Binding and Uptake of Ligands by Scavenger Receptors	42	9 (22.5%)	7.98E-04
Vesicle-mediated transport	Scavenging of heme from plasma	12	5 (41.7%)	1.12E-03
Vesicle-mediated transport	Formation of annular gap junctions	9	4 (44.4%)	3.35E-03
Vesicle-mediated transport	Gap junction degradation	10	4 (40.0%)	4.98E-03
Vesicle-mediated transport	Gap junction trafficking	12	4 (33.3%)	9.01E-03
Vesicle-mediated transport	Scavenging by Class F Receptors	6	3 (50.0%)	9.01E-03
Vesicle-mediated transport	Membrane Trafficking	580	44 (7.7%)	9.94E-03
Vesicle-mediated transport	Gap junction trafficking and regulation	14	4 (28.6%)	1.46E-02
Vesicle-mediated transport	ER to Golgi Anterograde Transport	134	14 (10.8%)	1.76E-02
Extracellular matrix organization	Extracellular matrix organization	295	17 (5.9%)	4.84E-01
	no sub-pathways detected			
Disease	Disease	514	39 (7.8%)	1.25E-02
Disease	Deregulated CDK5 triggers multiple neurodegenerative pathways in Alzheimer,s disease models	18	7 (38.9%)	1.30E-04

(table continued) Cluster	Pathway	Set size	Amount DEGs (%)	q-value
Disease	Neurodegenerative Diseases	18	7 (38.9%)	1.30E-04
Disease	Signaling by RAS mutants	37	8 (22.2%)	1.79E-03
Disease	Signaling by moderate kinase activity BRAF mutants	39	8 (21.1%)	2.55E-03
Disease	Paradoxical activation of RAF signaling by kinase inactive BRAF	39	8 (21.1%)	2.55E-03
Disease	Oncogenic MAPK signaling	64	10 (15.9%)	4.98E-03
Disease	Signaling by high-kinase activity BRAF mutants	35	7 (20.6%)	5.63E-03
Disease	Uptake and function of anthrax toxins	12	4 (33.3%)	9.01E-03
Disease	Entry of Influenza Virion into Host Cell via Endocytosis	2	2 (100.0%)	9.76E-03
Disease	Signaling by BRAF and RAF fusions	61	9 (15.0%)	1.00E-02
Immune System	Immune System	1950	124 (6.5%)	2.66E-03
Immune system	Neutrophil degranulation	497	60 (12.3%)	1.31E-09
Immune system	Innate Immune System	1309	102 (8.0%)	5.36E-06
Immune System	Regulation of Complement cascade	27	6 (22.2%)	7.91E-03
Immune System	MAP2K and MAPK activation	39	7 (18.4%)	9.47E-03
Immune System	Activation of C3 and C5	7	3 (42.9%)	1.35E-02
Immune System	Antigen Presentation: Folding, assembly and peptide loading of class I MHC	24	5 (20.8%)	1.95E-02
Immune System	MHC class II antigen presentation	60	8 (13.6%)	2.56E-02
Immune system	ISG15 antiviral mechanism	34	5 (16.7%)	4.22E-02
Immune system	Antiviral mechanism by IFN-stimulated genes	34	5 (16.7%)	4.22E-02
Muscle contraction	Muscle contraction	198	15 (7.7%)	1.66E-01
Muscle contraction	Smooth Muscle Contraction	36	9 (25.7%)	2.95E-04
Metabolism of proteins	Metabolism of proteins	1506	145 (9.9%)	2.77E-15
Metabolism of proteins	L13a-mediated translational silencing of Ceruloplasmin expression	129	37 (32.2%)	1.21E-18
Metabolism of proteins	3, -UTR-mediated translational regulation	129	37 (32.2%)	1.21E-18
Metabolism of proteins	GTP hydrolysis and joining of the 60S ribosomal subunit	129	37 (31.9%)	1.42E-18
Metabolism of proteins	Cap-dependent Translation Initiation	137	37 (30.1%)	1.01E-17
Metabolism of proteins	Eukaryotic Translation Initiation	137	37 (30.1%)	1.01E-17
Metabolism of proteins	Translation	177	40 (24.7%)	7.20E-16
Metabolism of proteins	Formation of a pool of free 40S subunits	117	31 (29.5%)	1.31E-14
Metabolism of proteins	Eukaryotic Translation Elongation	108	29 (29.9%)	6.72E-14
Metabolism of proteins	Peptide chain elongation	103	28 (30.4%)	1.17E-13
Metabolism of proteins	Eukaryotic Translation Termination	106	28 (29.5%)	2.75E-13
Metabolism of proteins	SRP-dependent cotranslational protein targeting to membrane	126	30 (26.3%)	8.16E-13
Metabolism of proteins	Ribosomal scanning and start codon recognition	69	22 (35.5%)	2.37E-12
Metabolism of proteins	Translation initiation complex formation	70	22 (35.5%)	2.37E-12
Metabolism of proteins	Activation of the mRNA upon binding of the cap-binding complex and eIFs, and subsequent binding to 43S	71	22 (34.9%)	3.31E-12
Metabolism of proteins	Cytosolic tRNA aminoacylation	26	14 (58.3%)	1.76E-11
Metabolism of proteins	UCH proteinases	107	26 (25.2%)	9.30E-11
Metabolism of proteins	tRNA Aminoacylation	45	16 (38.1%)	1.46E-09
Metabolism of proteins	Formation of the ternary complex, and subsequently, the 43S complex	61	18 (32.7%)	1.69E-09
Metabolism of proteins	Amyloid fiber formation	83	18 (22.0%)	1.32E-06
Metabolism of Proteins	Ub-specific processing proteases	225	29 (13.1%)	1.94E-05
Metabolism of Proteins	Formation of tubulin folding intermediates by CCT/TriC	26	8 (30.8%)	2.09E-04

(table continued) Cluster	Pathway	Set size	Amount DEGs (%)	q-value
Metabolism of Proteins	Deubiquitination	302	32 (10.8%)	2.23E-04
Metabolism of proteins	Asparagine N-linked glycosylation	283	30 (10.8%)	3.80E-04
Metabolism of Proteins	Cooperation of Prefoldin and TriC/CCT in actin and tubulin folding	34	8 (24.2%)	1.05E-03
Metabolism of Proteins	Prefoldin mediated transfer of substrate to CCT/TriC	29	7 (25.0%)	1.94E-03
Metabolism of proteins	N-glycan trimming in the ER and Calnexin/Calreticulin cycle	34	7 (21.2%)	4.98E-03
Metabolism of Proteins	Folding of actin by CCT/TriC	10	4 (40.0%)	4.98E-03
Metabolism of Proteins	Protein methylation	20	5 (25.0%)	9.76E-03
Metabolism of proteins	Synthesis of substrates in N-glycan biosythesis	63	9 (14.3%)	1.35E-02
Metabolism of Proteins	Post-chaperonin tubulin folding pathway	23	5 (21.7%)	1.69E-02
Metabolism of proteins	Post-translational protein modification	1026	68 (6.7%)	1.85E-02
Metabolism of proteins	Mitochondrial protein import	63	8 (12.7%)	3.60E-02
Metabolism of proteins	Protein folding	106	11 (10.7%)	3.80E-02
Metabolism of proteins	Transport to the Golgi and subsequent modification	165	15 (9.3%)	4.01E-02
Metabolism of proteins	Biosynthesis of the N-glycan precursor (dolichol lipid-linked oligosaccharide, LLO) and transfer to a nascent protein	78	9 (11.5%)	4.22E-02
Metabolism of proteins	Mitochondrial tRNA aminoacylation	23	4 (19.0%)	4.97E-02
Metabolism of proteins	Cooperation of PDCL (PhLP1) and TRiC/CCT in G-protein beta folding	44	6 (14.0%)	4.99E-02
Cellular responses to stress	Cellular responses to stress	393	36 (9.3%)	1.25E-03
Cellular responses to stress	Senescence-Associated Secretory Phenotype (SASP)	113	17 (15.3%)	2.42E-04
Cellular responses to stress	Oxidative Stress Induced Senescence	129	17 (13.5%)	1.02E-03
Cellular responses to stress	HSF1 activation	31	7 (22.6%)	3.51E-03
Cellular responses to stress	HSF1-dependent transactivation	37	7 (19.4%)	7.71E-03
Cellular responses to stress	Attenuation phase	29	6 (21.4%)	9.01E-03
Cellular responses to stress	Cellular Senescence	192	19 (10.1%)	9.76E-03
Cellular responses to stress	Detoxification of Reactive Oxygen Species	36	6 (17.1%)	2.28E-02
Hemostasis	Hemostasis	693	43 (6.4%)	1.37E-01
Hemostasis	Platelet degranulation	133	22 (16.9%)	5.27E-06
Hemostasis	Response to elevated platelet cytosolic Ca2+	138	22 (16.3%)	9.72E-06
Hemostasis	Platelet activation, signaling and aggregation	283	28 (10.0%)	1.76E-03
Hemostasis	p130Cas linkage to MAPK signaling for integrins	16	4 (26.7%)	1.83E-02
Developmental Biology	Developmental Biology	748	49 (6.6%)	7.98E-02
Developmental Biology	Recycling pathway of L1	29	6 (21.4%)	9.01E-03
Developmental biology	Axon guidance	487	38 (7.9%)	1.12E-02
Developmental biology	EPH-Ephrin signaling	78	9 (11.5%)	4.22E-02
Developmental biology	Semaphorin interactions	68	8 (11.9%)	4.80E-02
Developmental biology	RAF/MAP kinase cascade	200	17 (8.7%)	4.83E-02
Cell-Cell communication	Cell-Cell communication	131	8 (6.2%)	5.30E-01
	no sub-pathways detected			
Programmed Cell Death	Programmed Cell Death	125	18 (14.8%)	2.42E-04
Programmed Cell Death	Apoptosis	122	18 (15.1%)	1.84E-04
Programmed Cell Death	Activation of BAD and translocation to mitochondria	15	6 (40.0%)	3.80E-04
Programmed Cell Death	Apoptotic execution phase	53	10 (19.2%)	1.25E-03
Programmed Cell Death	Apoptotic cleavage of cellular proteins	39	7 (18.4%)	9.47E-03
Programmed Cell Death	Activation of BH3-only proteins	31	6 (20.0%)	1.17E-02

(table continued) Cluster	Pathway	Set size	Amount DEGs (%)	q-value
Programmed Cell Death	Intrinsic Pathway for Apoptosis	44	7 (16.3%)	1.71E-02
Programmed Cell Death	Breakdown of the nuclear lamina	3	2 (66.7%)	2.30E-02
Programmed Cell Death	Regulation of Apoptosis	4	2 (50.0%)	4.01E-02
Transport of small molecules	(Transmembrane) transport of small molecules	628	21 (3.4%)	1.00E+00
Transport of small molecules	Erythrocytes take up oxygen and release carbon dioxide	9	3 (33.3%)	2.54E-02
Signal transduction	Signal transduction	2538	105 (4.2%)	1.00E+00
Signal transduction	RHO GTPases activate PKNs	96	23 (24.2%)	3.61E-09
Signal transduction	RHO GTPase Effectors	299	38 (12.9%)	9.86E-07
Signal transduction	Activated PKN1 stimulates transcription of AR (androgen receptor) regulated genes KLK2 and KLK3	70	16 (23.2%)	2.73E-06
Signal transduction	Formation of the beta-catenin:TCF transactivating complex	95	17 (18.3%)	3.00E-05
Signal transduction	RHO GTPases activate PAKs	23	8 (34.8%)	8.87E-05
Signal transduction	Signaling by Wnt	286	31 (11.1%)	1.84E-04
Signal transduction	Signaling by Rho GTPases	434	41 (9.7%)	2.42E-04
Signal transduction	VEGFR2 mediated vascular permeability	29	8 (27.6%)	4.49E-04
Signal transduction	DARPP-32 events	27	7 (26.9%)	1.26E-03
Signal transduction	RHO GTPases activate IQGAPs	13	5 (38.5%)	1.62E-03
Signal transduction	TCF dependent signaling in response to WNT	195	20 (10.6%)	5.47E-03
Signal transduction	CaMK IV-mediated phosphorylation of CREB	5	3 (60.0%)	5.48E-03
Signal transduction	Cam-PDE 1 activation	6	3 (50.0%)	9.01E-03
Signal Transduction	GRB2:SOS provides linkage to MAPK signaling for Integrins	16	4 (26.7%)	1.83E-02
Signal transduction	VEGFA-VEGFR2 Pathway	282	23 (8.3%)	3.34E-02
Signal transduction	Calmodulin induced events	28	5 (17.9%)	3.35E-02
Signal transduction	CaM pathway	28	5 (17.9%)	3.35E-02
Signal transduction	MAPK family signaling cascades	240	20 (8.5%)	3.65E-02
Signal transduction	Ca-dependent events	30	5 (16.7%)	4.22E-02
Signal transduction	Signaling by VEGF	290	23 (8.0%)	4.25E-02
Signal transduction	Signaling by Insulin receptor	278	22 (8.1%)	4.72E-02
Signal Transduction	SHC1 events in EGFR signaling	200	17 (8.7%)	4.83E-02
Signal Transduction	SOS-mediated signalling	200	17 (8.7%)	4.83E-02
Signal Transduction	GRB2 events in EGFR signaling	200	17 (8.7%)	4.83E-02
Signal transduction	Beta-catenin independent WNT signaling	97	10 (10.6%)	4.84E-02
Neuronal System	Neuronal System	351	12 (3.5%)	9.97E-01
Neuronal System	CREB phosphorylation through the activation of CaMKII	17	6 (35.3%)	7.85E-04
Neuronal System	Ras activation uopn Ca2+ infux through NMDA receptor	19	6 (31.6%)	1.37E-03
Neuronal System	Activation of CaMK IV	4	3 (75.0%)	2.60E-03
Neuronal System	CREB phosphorylation through the activation of CaMKK	6	3 (50.0%)	9.01E-03
Neuronal System	CREB phosphorylation through the activation of Ras	29	6 (20.7%)	1.00E-02
Neuronal System	Post NMDA receptor activation events	37	6 (16.2%)	2.77E-02
Neuronal System	Activation of NMDA receptor upon glutamate binding and postsynaptic events	41	6 (14.6%)	4.22E-02
Gene expression	Gene Expression	1755	115 (6.8%)	1.22E-03
Gene expression	Nonsense Mediated Decay (NMD) independent of the Exon Junction Complex (EJC)	109	29 (29.9%)	6.72E-14
Gene expression	Nonsense Mediated Decay (NMD) enhanced by the Exon Junction Complex (EJC)	122	29 (26.6%)	1.47E-12
Gene expression	Nonsense-Mediated Decay (NMD)	122	29 (26.6%)	1.47E-12

(table continued) Cluster	Pathway	Set size	Amount DEGs (%)	q-value
Gene expression	RNA Polymerase I Promoter Opening	66	16 (24.6%)	1.31E-06
Gene expression	DNA methylation	68	16 (23.9%)	1.92E-06
Gene expression	SIRT1 negatively regulates rRNA Expression	72	16 (22.9%)	3.24E-06
Gene expression	PRC2 methylates histones and DNA	77	16 (21.3%)	8.17E-06
Gene expression	ERCC6 (CSB) and EHMT2 (G9a) positively regulate rRNA expression	79	16 (20.5%)	1.31E-05
Gene expression	B-WICH complex positively regulates rRNA expression	96	17 (18.3%)	3.00E-05
Gene expression	RNA Polymerase I Chain Elongation	94	16 (17.4%)	1.01E-04
Gene expression	Transcriptional regulation by small RNAs	108	17 (15.9%)	1.69E-04
Gene expression	Positive epigenetic regulation of rRNA expression	111	17 (15.7%)	1.84E-04
Gene expression	NoRC negatively regulates rRNA expression	110	16 (14.8%)	5.90E-04
Gene expression	RNA Polymerase I Promoter Clearance	113	16 (14.4%)	7.82E-04
Gene expression	Negative epigenetic regulation of rRNA expression	113	16 (14.4%)	7.82E-04
Gene expression	RNA Polymerase I Transcription	115	16 (14.2%)	9.05E-04
Gene expression	Gene Silencing by RNA	134	17 (13.0%)	1.50E-03
Gene expression	TP53 Regulates Metabolic Genes	86	12 (14.1%)	4.91E-03
Gene expression	Epigenetic regulation of gene expression	154	17 (11.3%)	5.69E-03
Gene expression	Regulation of mRNA stability by proteins that bind AU-rich elements	40	7 (18.9%)	8.82E-03
Gene expression	AUF1 (hnRNP D0) binds and destabilizes mRNA	7	3 (50.0%)	9.01E-03
Gene expression	RNA Polymerase I, RNA Polymerase III, and Mitochondrial Transcription	153	16 (10.6%)	1.23E-02
Gene expression	Deadenylation of mRNA	25	5 (21.7%)	1.69E-02
Gene expression	mRNA Splicing - Major Pathway	185	17 (9.7%)	2.15E-02
Gene expression	mRNA Splicing	193	17 (9.2%)	2.99E-02
DNA repair	DNA repair	323	22 (7.0%)	1.85E-01
DNA repair	Nonhomologous End-Joining (NHEJ)	71	16 (22.9%)	3.24E-06
DNA repair	Recruitment and ATM-mediated phosphorylation of repair and signaling proteins at DNA double strand breaks	77	16 (20.8%)	1.13E-05
DNA repair	DNA Double Strand Break Response	78	16 (20.5%)	1.31E-05
DNA repair	Processing of DNA double-strand break ends	100	16 (16.2%)	2.22E-04
DNA repair	Homology Directed Repair	142	17 (12.1%)	2.99E-03
DNA repair	HDR through Homologous Recombination (HR) or Single Strand Annealing (SSA)	136	16 (11.9%)	4.95E-03
DNA repair	DNA Double-Strand Break Repair	171	18 (10.7%)	7.69E-03
Chromatin organization	Chromatin organization	174	17 (6.3%)	3.83E-01
Chromatin organization	HDMs demethylate histones	52	14 (27.5%)	1.87E-06
Chromatin organization	RMTs methylate histone arginines	74	15 (20.3%)	3.00E-05
Chromatin organization	PKMTs methylate histone lysines	73	14 (19.7%)	8.40E-05
Chromatin organization	HDACs deacetylate histones	94	14 (14.9%)	1.26E-03
Chromatin organization	HATs acetylate histones	143	16 (11.3%)	7.91E-03
Cell Cycle	Cell Cycle	551	42 (7.7%)	1.00E-02
Cell Cycle	Deposition of new CENPA-containing nucleosomes at the centromere	43	15 (34.9%)	2.19E-08
Cell Cycle	Nucleosome assembly	43	15 (34.9%)	2.19E-08
Cell Cycle	Meiotic synapsis	48	14 (29.8%)	6.97E-07
Cell Cycle	G2/M DNA damage checkpoint	97	20 (20.8%)	7.11E-07
Cell Cycle	Meiotic recombination	65	16 (25.0%)	1.06E-06

(table continued) Cluster	Pathway	Set size	Amount DEGs (%)	q-value
Cell Cycle	Condensation of Prophase Chromosomes	77	17 (22.4%)	2.08E-06
Cell Cycle	Packaging Of Telomere Ends	54	14 (25.9%)	3.43E-06
Cell Cycle	Meiosis	79	16 (20.8%)	1.13E-05
Cell Cycle	G2/M Checkpoints	121	20 (16.7%)	1.94E-05
Cell Cycle	Mitotic Prophase	143	22 (15.5%)	2.07E-05
Cell Cycle	Telomere Maintenance	85	16 (19.0%)	3.28E-05
Cell Cycle	Cell Cycle Checkpoints	158	21 (13.5%)	2.30E-04
Cell Cycle	Chromosome Maintenance	113	16 (14.3%)	8.21E-04
Cell Cycle	Chk1/Chk2(Cds1) mediated inactivation of Cyclin B:Cdk1 complex	12	5 (41.7%)	1.12E-03
Cell Cycle	Regulation of PLK1 Activity at G2/M Transition	90	13 (14.8%)	2.12E-03
Cell Cycle	Loss of Nlp from mitotic centrosomes	72	10 (14.3%)	9.15E-03
Cell Cycle	Loss of proteins required for interphase microtubule organization from the centrosome	72	10 (14.3%)	9.15E-03
Cell Cycle	M Phase	267	24 (9.2%)	1.05E-02
Cell Cycle	AURKA Activation by TPX2	75	10 (13.7%)	1.16E-02
Cell Cycle	Recruitment of mitotic centrosome proteins and complexes	82	10 (12.5%)	2.01E-02
Cell Cycle	Centrosome maturation	82	10 (12.5%)	2.01E-02
Cell Cycle	G2/M Transition	136	14 (10.5%)	2.06E-02
Cell Cycle	Mitotic G2-G2/M phases	138	14 (10.4%)	2.30E-02
Cell Cycle	Optineurin and Myosin Phosphatase Negatively Regulate PLK1	3	2 (66.7%)	2.30E-02
Cell Cycle	Cell Cycle, Mitotic	468	35 (7.6%)	2.49E-02
Organelle biogenesis and maintenance	Organelle biogenesis and maintenance	310	17 (5.6%)	5.77E-01
Organelle biogenesis and maintenance	Anchoring of the basal body to the plasma membrane	100	12 (12.2%)	1.21E-02
